# Systematic chromatin state comparison of epigenomes associated with diverse properties including sex and tissue type

**DOI:** 10.1038/ncomms8973

**Published:** 2015-08-18

**Authors:** Angela Yen, Manolis Kellis

**Affiliations:** 1 Electrical Engineering and Computer Science Department, Computer Science and Artificial Intelligence Laboratory, MIT, 32 Vassar Street, 32D-524, Cambridge, Massachusetts 02139, USA; 2 Broad Institute of MIT and Harvard, Cambridge, Massachusetts 02142, USA

## Abstract

Epigenomic data sets provide critical information about the dynamic role of chromatin states in gene regulation, but a key question of how chromatin state segmentations vary under different conditions across the genome has remained unaddressed. Here we present ChromDiff, a group-wise chromatin state comparison method that generates an information-theoretic representation of epigenomes and corrects for external covariate factors to better isolate relevant chromatin state changes. By applying ChromDiff to the 127 epigenomes from the Roadmap Epigenomics and ENCODE projects, we provide novel group-wise comparative analyses across sex, tissue type, state and developmental age. Remarkably, we find that distinct sets of epigenomic features are maximally discriminative for different group-wise comparisons, in each case revealing distinct enriched pathways, many of which do not show gene expression differences. Our methodology should be broadly applicable for epigenomic comparisons and provides a powerful new tool for studying chromatin state differences at the genome scale.

## Abstract (web summary)

In contrast to genetic information, epigenetic state varies greatly under different conditions. Here, the authors develop ChromDiff and apply it to ENCODE and Roadmap Epigenomics datasets to find chromatin state differences associated with sex, tissue, and developmental age.

## Graphical abstract

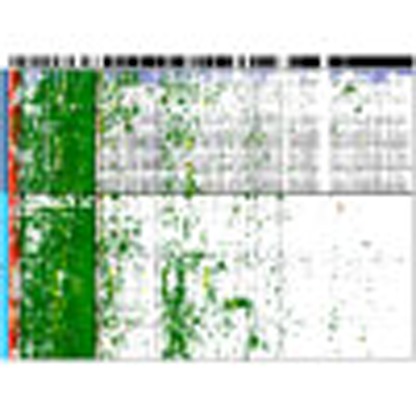

Epigenomic data sets provide a rich resource for understanding genome activity across both genes and regulatory regions in response to developmental, environmental or genetic signals. Epigenomic marks, including histone modifications and DNA methylation, have been shown to be highly dynamic across cell types^1^,^2^,^3^. Furthermore, epigenetic differences have been strongly associated with changes in mammalian development^4^,^5^, as well as gene activation and repression patterns across cell types^6^,^7^,^8^,^9^. Epigenomic signatures have also resulted in the identification of new regulatory elements and functional annotations, even in regions that fall in unconserved genetic sequences^10^,^11^,^12^.

In addition to cell type differences, comparative epigenomics analyses have been applied across individuals, disease status and species. Studies of natural epigenomic variation across individuals have shown wide-spread differences across individuals of different genotypes, and between the two alleles of the same individual^13^,^14^. Epigenomic comparisons across disease and control samples have been linked to differences in disease manifestation in monozygotic twins^15^, while ongoing efforts such as the International Cancer Genome Consortium (ICGC)^16^ aim to better understand the role of epigenomic alterations in cancer. Comparative epigenomics analysis across species has also proved informative, identifying conserved and distinct epigenetic marks, and tools such as the Comparative Epigenome Browser^12^,^17^,^18^,^19^ (CEpBrowser) allow for direct exploration of multi-species epigenome comparisons^20^.

As our understanding of epigenomics has progressed, previous methods have leveraged histone combinations to partition the epigenome into various chromatin states, such as ChromHMM^21^, Segway^22^ and HMMSeg^23^. The resulting analyses enabled by chromatin state analysis has provided fruitful findings about epigenomic variation and lineage specification^24^,^25^,^26^,^27^,^28^,^29^. However, no methods have yet been developed to enable group-wise chromatin state comparisons based on these combinatorial segmentations.

Comparative epigenomic analyses initially focused on peak-calling, enrichments, domains or comparisons for a single histone modification with various normalization and modelling approaches^30^,^31^,^32^,^33^,^34^. As the availability of data increased rapidly in recent years, methods tackling combinatorial approaches to histone modification data to identify patterns across many histone marks for one biological condition or sample have been developed^35^,^36^,^37^,^38^, including the aforementioned segmentation methods^21^,^22^,^23^.

However, scalable combinatorial methods to directly discover patterns between chromatin state changes and biological conditions are still limited. MultiGPS addresses the analogous question of comparing transcription factor binding chromatin immunoprecipitation sequencing (ChIP-Seq) experiments across groups^39^, and therefore tailors the approach to punctate signals that are not relevant for histone mark data. To our knowledge, only one method, differential principal component analysis (dPCA)^40^, compares epigenomic signal across multiple histone marks under multiple conditions; it does so by performing PCA analysis on the differences of the replicate averages. While dPCA has been shown to be useful, it is constrained by the limitations of PCA analysis, such as sensitivity to scaling the data. Furthermore, dPCA does not provide any options to correct for external covariate factors. Covariate correction is a crucial part of comparative analysis when using data sets with variation due to batch effects, donor variability, sample differences and experimental differences. In addition, the importance of covariate correction will only increase in coming years, with the release of more public and resource data sets that will increase statistical power but will also be generated in less controlled circumstances. Last, dPCA compares the histone mark signal based on differences in means, but does not take advantage of existing advanced techniques that interpret combinatorial histone mark signals into segmentations based on Hidden Markov Models (HMMs) or bayesian networks.

In this paper, we propose a highly scalable method, ChromDiff, for directly discovering potential relationships between chromatin states, genes and biological conditions; in doing so, ChromDiff generates a novel information-theoretic representation for epigenetic information and employs covariate correction to enable large-scale analysis of samples while controlling for a wide variety of circumstances, including batch effects and donor variability. As a result, ChromDiff is a general statistical pipeline for comparing combinatorial chromatin states of groups of epigenomes, which we then apply to leverage the breadth of data from the Roadmap Epigenomics and ENCODE projects^10^,^41^ and the diversity of chromatin state annotations provided by ChromHMM^21^,^41^.

Specifically, by utilizing the chromatin state annotation for every epigenome, we use these discrete states to quickly compare any subset of epigenomes to one another through a probabilistic representation of the chromatin states that builds on information theory. By utilizing chromatin states that were jointly learned over all epigenomes, we are able to use a general model, but apply it to many specific biological questions. To account for various differences in sample and data generation, we also utilize the metadata of the epigenomes to correct for covariate factors, thereby better isolating differences due to a single biological attribute. This covariate correction allows ChromDiff to leverage the same set of epigenomic data for various, specific biological conditions, while controlling for other variables. Furthermore, ChromDiff is compatible with any general chromatin state segmentation, regardless of the method behind it, which makes it amenable to various existing methods, including ChromHMM^21^, Segway^22^ and HMMSeg^23^, as well as future methods that are yet to be developed.

Similar to other methods, ChromDiff produces sets of regions with epigenomic differences across conditions. However, our method additionally utilizes group-wise comparisons to gain statistical power, while building on a general chromatin state model segmentation. As a pipeline, ChromDiff also provides additional features of gene set enrichment calculations and gene expression comparisons. Furthermore, ChromDiff clusters the distinguishing genomic regions into groups that exhibit similar epigenomic signatures, thereby highlighting clusters with distinct gene set enrichment and gene expression patterns. These results suggest that the identified clusters may share regulatory mechanisms and functional pathways. In this way, ChromDiff provides novel, thorough insights on the complex relationship between these general chromatin states, biological attributes and specific clusters of genes. This method, therefore, not only enables the identification of genomic regions relevant to an epigenomic comparison based on group-wise differences, but also provides a global understanding of how chromatin states are involved in a wide variety of biological situations.

To demonstrate the power of our method, we apply ChromDiff to identify genes and chromatin states that differentiate epigenomes across donor sex, tissue type, sample state and donor developmental age. The results reveal that distinct types of epigenomic features vary with different biological properties and strongly validate our statistical approach. In addition, our specific comparisons result in new biological insights on the types of epigenomic features and pathways that underlie each of our comparisons. More generally, we believe our method will be broadly applicable to new epigenomic data sets, and that epigenomic comparisons across multiple marks and multiple samples will be widely used to uncover the molecular processes underlying cellular differentiation, gene regulation and human disease.

## Results

### Comparison of epigenomic features

**Figure 1:**
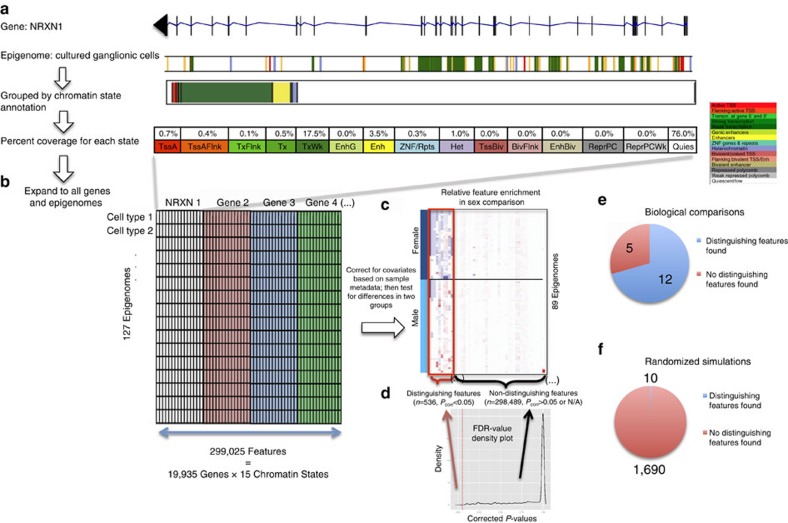
A novel method for comparative analysis of epigenomic groups. (**a**) Starting with a single gene (NRXN1) and epigenome (cultured ganglionic cells), we represent the epigenome as the per cent coverage of each chromatin state at that gene. (**b**) Then, we repeat the process for all 127 epigenomes and 19,935 protein-coding genes, resulting in a matrix of 127 epigenomes by 299,025 features. (**c**) After normalizing and correcting each column in the matrix for covariate factors, we compare the female and male epigenomes of the original 127 epigenomes to identify features that exhibit different behaviour in female and male epigenomes. (**d**) The density plot of corrected *P* values from all features shows 536 out 299,025 features that significantly differ between the 2 groups. (**e**) Of the real biological comparisons that we tried, we found distinguishing epigenomic differences over 70% of the time. (**f**) Distinguishing features were found for randomized groupings only 10 out of 1,700 times or <1% of the time.

To capture epigenomic differences between groups of epigenomes, we focus on the set of chromatin states associated with each protein-coding gene (Fig. 1), while generating an information-theoretic encoding of these chromatin states and correcting for external factors to isolate differences due to the comparison. We leverage the multiple samples available in each pairwise group comparison to evaluate the statistical significance of such recurrent changes, and the multiple genes to evaluate the statistical significance of biological pathways. However, our methods are generally and broadly applicable to various regulatory genomic regions, beyond the gene-centric approach taken here.

Specifically, we define epigenomic features by calculating the probability of chromatin state assignment for each gene across each epigenome, integrated over the body of that gene (Fig. 1). For example, we apply our method to gene *NRXN1* in neurosphere cultured cells (Fig. 1a), based on the 15-state ChromHMM annotation of the Roadmap Epigenomics project^41^. First, we identify the probability that *NRXN1* should be assigned to each of 15 chromatin states, integrated over the entire length on the gene body, resulting in 15 different features for *NRXN1*. This encoding is based on information theory, as we retain the probability distribution of each chromatin state within each gene and sample type. Therefore, this representation drastically reduces the dimension of the chromatin state data while preserving the information necessary to calculate important information theory metrics, including the entropy of each gene and sample type, as well as the divergence between one gene and another gene or background. As information theory has been shown to have applications to fields as diverse as signal processing, neurobiology, machine learning and cryptography, it provides a theoretical foundation for our method.

ChromDiff recalculates this encoding for every gene and every epigenome, resulting in a matrix of 299,025 features (columns) and 127 epigenomes (rows) (Fig. 1b). We then utilize logistic regression to correct for feature covariates including production centre, sex of donor, sample state (solid or liquid) and sample type (cell line, primary cell, tissue and so on) by setting the value of each covariate factor that we are not testing to be the response residuals from the logistic regression model (Fig. 1c). This step is crucial due to the wide variety of differences among the epigenomes; by controlling for variables that we are not currently investigating, ChromDiff is better able to identify genes with chromatin state changes that specifically correspond to the current comparison. As a result, each feature value indicates whether that gene is annotated as that chromatin state more or less often than expected, after correcting for covariates.

Finally, our pipeline uses these corrected feature values to recognize significant differences between two groups of samples, using a non-parametric Mann–Whitney–Wilcoxon test, Student's *t*-test or *F*-test, with correction for multiple hypothesis testing using Bonferroni, Benjamini–Hochberg or Benjamini–Yekuteli multiple hypothesis correction. (In this example, we used male and female samples with the Mann-Whitney-Wilcoxon test and Benjamini-Hochberg correction.) Based on these statistical results, ChromDiff reports all features (chromatin state and gene combinations) that are significantly different between the two groups at a corrected *P* value cutoff of *P*<0.05 (Fig. 1d).

Though ChromDiff can be applied to any genomic region, we focused on gene bodies here, as they make our methodology and results easier to validate and interpret. This approach allowed us to incorporate into the ChromDiff pipeline multiple tools for downstream analysis of the resulting genes that are found to show epigenomic differences in our comparisons. First, to recognize the biological processes associated with epigenomic differences, we studied the ontology enrichments of genes associated with different significant features (see ‘Gene set enrichment calculations'). Second, for each comparison, we compared the expression level of genes with significant epigenomic features, to evaluate whether epigenomic differences are also reflected in gene expression differences. Last, we used hierarchical clustering to recognize clusters of features and genes that show consistent differences between samples (see Methods); using these clusters, we are able to find cluster-specific gene sets with specific gene set enrichment and expression behaviour.

In addition to developing our ChromDiff pipeline, we also applied it to the epigenomic data from the Roadmap Epigenomics project; here we present the results by applying ChromDiff for 17 group-wise comparisons (Supplementary Table 1). Specifically, we used the segmentation of the Roadmap Epigenomics 15-state ChromHMM model, the statistical test of the non-parametric Mann–Whitney–Wilcoxon test, and the multiple hypothesis correction of Benjamini–Hochberg false discovery rate (FDR) correction (see Methods). In total, we found significant epigenomic features in over 70% of the biological groupings we tested (12/17) (Fig. 1e, Supplementary Table 1).

To validate our results and methodology, we performed randomized simulations that quantified how likely we would have obtained results with randomized data. By repeatedly shuffling the epigenomes for each of the 17 biological comparisons we tested (see ‘Randomized simulations'), we found the shuffled groups resulted in ‘significant features' <1% of the time (10/1,700 simulations) (Fig. 1f). This suggests that ChromDiff is able to pick up a real, biologically meaningful signal from our biological comparisons (Fig. 1e). Furthermore, if ChromDiff operated with a bias towards certain chromatin states or gene sizes, this should become evident in the results from our simulations. However, in practice, we found no consistent bias towards any chromatin state (Supplementary Fig. 1a) or gene size (Supplementary Fig. 2a) in results from our randomized simulations. This provides confidence that the distribution of chromatin states (Supplementary Fig. 1b) and gene sizes (Supplementary Fig. 2b) in our real results is based on real biological signal; for example, some brain-specific genes have been shown to be long^42^ and three of our four comparisons that identified the longest genes on average involved brain epigenomes.

### Applications

For the remainder of the paper, we will present the results from four of the epigenomic comparisons based on donor sex, sample tissue type, sample state and donor developmental age. Then, we demonstrate how ChromDiff presents a complementary approach to differential gene expression analysis, and we show that ChromDiff outperforms the current tool for epigenomic group-wise comparative analysis on the Roadmap Epigenomics data set^41^.

### Polycomb repression distinguishes female epigenomes

**Figure 2:**
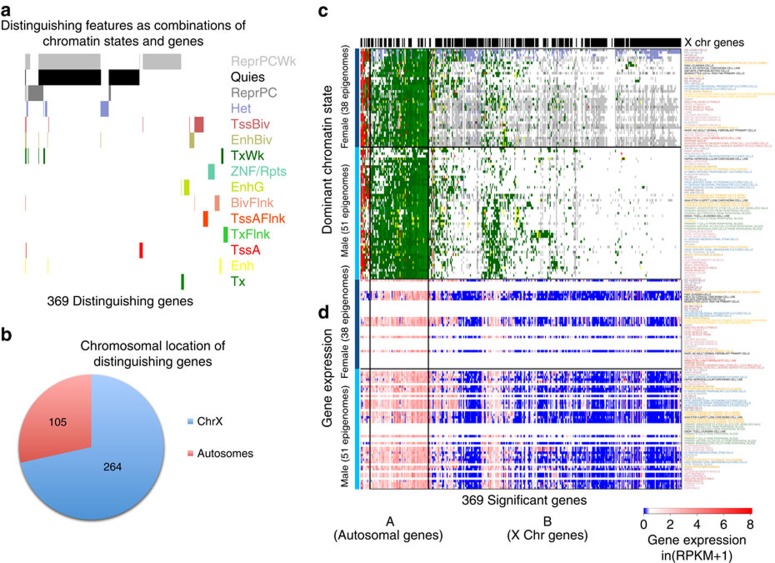
X chromsome inactivation distinguishes male and female samples. Comparison of male and female epigenomes identifies (**a**) 536 distinguishing features that are associated with 369 genes and all 15 chromatin states, where (**b**) 264 of the 369 genes are located on the X chromosome. (**c**) 124 of the identified X chromsome genes are mainly quiescent in male samples but weakly repressed or heterochromatic in female cell types (mostly in cluster B), while 56 genes are transcribed in female and male samples (mostly autosomal genes in cluster A), shown here by the most abundant chromatin state for these genes. (**d**) Expression data for these genes (when available) confirm similar expression levels between male and female samples, as suggested by the chromatin state annotations.

In our first comparison, we sought epigenomic differences between male and female samples. We found 536 significant epigenomic features (gene–chromatin state combinations) distinguishing male from female samples, which we will call ‘distinguishing features'; these features correspond to 369 genes (that we will refer to as ‘distinguishing genes') and encompass all 15 chromatin states (Fig. 2a). Most distinguishing genes are only associated with 1 feature (only a single chromatin state is significantly different), with the exception of 133 genes that exhibit significant differences in multiple chromatin states, mostly quiescent and weak Polycomb repression (114 of 133 genes) (Fig. 2a).

Remarkably, over 70% of the distinguishing genes are located on the X chromosome (264 of the 369 genes) (Fig. 2b). Many of these chromosome X genes (124 of 264 genes) are primarily quiescent in male samples and primarily heterochromatic or Polycomb-repressed in female samples, as exemplified by many of the genes in cluster B in Fig. 2c, which visualizes the most abundant chromatin state for each distinguishing gene. For these 124 genes, the X chromosome location and epigenomic signature of Polycomb and heterochromatic repression in females are consistent with known mechanisms of X inactivation^43^. In addition, we see another epigenomic signature (exemplified by gene cluster A in Fig. 2c) at the 56 genes that are mostly transcribed in both females and males (31 of these 56 genes are autosomal). Supplementary Fig. 3 shows that many of these transcribed and autosomal genes are associated with changes in bivalent (TssBiv, EnhBiv and BivFlnk), enhancer (EnhG and Enh) and transcribed (TxFlnk and TxWk) regions. Overall, gene expression is largely unchanged between the female and male epigenomes at the distinguishing genes, despite the epigenomic differences (Fig. 2d), with only 2 out of the 368 distinguishing genes with expression data exhibiting significantly different expression levels. Again, this is consistent with X inactivation due to the allelic imbalance of X chromosomes for female and male donors.

### Brain and GI differences relate to neuronal genes

We next compared brain cells and tissues against gastrointestinal (GI) tissues, two of the anatomical groups for which we had the most epigenomic data. We found 10,455 distinguishing features, corresponding to 5,533 distinguishing genes. For visualization purposes, we have sampled down in this and future examples to 10,000 distinguishing features and their associated genes (see Methods).

**Figure 3:**
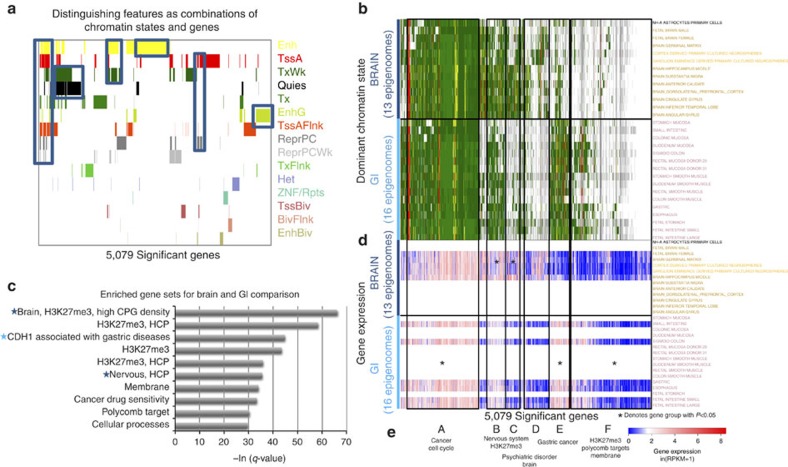
Transcriptional differences dominate brain and GI tissue comparison. Comparison of brain and gastrointestinal epigenomes reveal (**a**) coordinated chromatin state changes that co-occur within groups of genes, as well as cluster-specific transcriptional differences at associated genes based on (**b**) most abundant chromatin state and (**d**) gene expression data (when available). Five of the six identified gene groups have significantly different expression between brain and GI samples, with asterisks indicating *P*<0.05 based on the two-sided Mann–Whitney-Wilcoxon test. (**c**) Identified genes are enriched for brain- (dark blue stars) and gastric-specific (light blue stars) purposes and gene sets, as evidenced by the top 10 gene set annotations. (**e**) Genes in each epigenomic cluster contain different gene set annotations, such as cancer-related and cell cycle gene sets (cluster A), gastric-specific (cluster E) and brain-specific (cluster D) gene sets, genes related to the nervous system (cluster C), genes associated with histone marks (clusters C and F) and membrane genes (cluster F).

Over 40% (2,274 of 5,533 genes) of the genes distinguishing brain from GI tissues involve multiple chromatin states for each gene. Of the 5,079 genes associated with the 10,000 sampled features, 6 groups of genes emerge, representing genes with distinguishing features involving: (a) promoter and enhancer regions, (b) weakly transcribed and quiescent regions; (c) enhancer and weakly transcribed regions, (d) enhancer regions only, (e) Polycomb-repressed and active transcription start site regions, and (f) genetic enhancer regions (Fig. 3a, left to right, Supplementary Fig. 4). These results highlight the powerful ability of ChromDiff to identify relationships between chromatin states: these gene groups suggest combinations of chromatin states that act in coordinated ways to complement and/or reinforce one another.

In contrast to the sex-based comparison, the comparison of brain and GI tissues identifies many genes with significant expression differences. Specifically, in 18% of the discriminative genes (1,043/5,533), the most abundant chromatin state switched between mainly transcribed in one group (Tx, TxWk or TxFlnk) to primarily Polycomb-repressed or quiescent in the other group (ReprPC, ReprPCWk or Quies), as exemplified by gene clusters C, D, E and F (Fig. 3b). The majority of these switching genes (675/1,043) showed significant expression differences from RNA-Seq data (Fig. 3d). Overall, 40% of all distinguishing genes showed significantly different expression (2,236/5,507).

For many of the remaining genes, including those in gene cluster A (Fig. 3b), epigenomic differences did not involve the most abundant chromatin state. For example, in both brain and GI epigenomes, 86% of genes in cluster A are annotated as primarily transcribed or enhancer states (1,253/1,452 genes), but the majority of cluster A genes were identified based on features that did not involve transcription or enhancer states (1,177/1,452). This suggests that, in addition to dominant chromatin state switching, subtle but consistent epigenomic changes may also play an important role in distinguishing sample groups from one another.

Furthermore, our gene clustering based on epigenomic signal also revealed that different gene clusters mapped to varying gene set functions, ranging from brain-specific genes (clusters C and D), genes related to gastric cancer (cluster E), cell cycle genes (cluster A), Polycomb targets and genes marked by H3K27me3 (cluster F), and genes associated with cancer (clusters A, B, and E) (Fig. 3e, Supplementary Tables 2–7). The entire set of 5,533 distinguishing genes is enriched for genes known to be important for brain and GI function, including genes with brain-specific histone modifications and targets of CDH1, which has recently been shown to be associated with gastric cancer^44^,^45^ (Fig. 3c, Supplementary Table 8).

### Blood samples distinguished by enhancer activity differences

**Figure 4:**
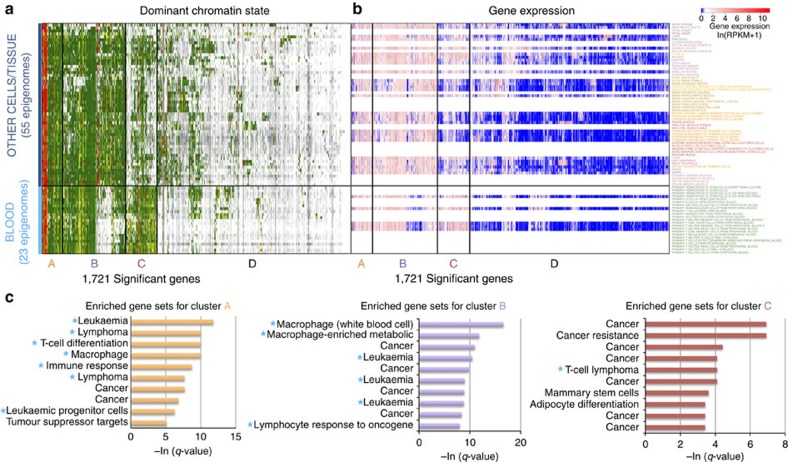
Epigenomic differences specific to blood samples lie at blood cancer genes. Comparison of blood epigenomes with other solid primary cells and tissues reveals distinguishing epigenomic activity, at genes marked by (**a**) transcriptional, enhancer, repressed and quiescent chromatin states, as shown by the most abundant chromatin state heatmap. The corresponding transcriptional activity can be seen in (**b**) gene expression profiles for the associated genes (with the genes in the same ordering as in **a**). (**c**) Genes in clusters A and B are enriched for blood-specific gene sets (light blue stars), such as gene sets relating to immune response, lymphoma, leukaemia and macrophage activity. On the other hand, cluster C is generally enriched for genes relating to cancer, while cluster D is largely enriched for membrane genes.

With the resources of various blood epigenomes, we compared chromatin states at gene bodies for the liquid samples (blood) against the solid samples (tissues and other primary cells). ChromDiff found 45,513 significant distinguishing features associated with 17,001 genes. The 10,000 sampled features and their associated 1,721 genes are largely dominated by transcription, enhancer, quiescent and repression states (Fig. 4a), and about 40% of the genes show expression differences (717/1,717 of sampled genes, 6,827/16,827 distinguishing genes) (Fig. 4b).

We find four main clusters of genes (Fig. 4a,b) that correspond to different gene set enrichments. Gene cluster A is characterized by strong enrichment for gene sets relating to immune response, T-cell differentiation and blood cancers, while cluster B is enriched for genes related to macrophage function and leukaemia (Fig. 4c). Cluster C is enriched for genes relating to general cancer development, and cluster D is enriched for membrane genes, likely due to blood cell-specific membrane function^46^,^47^ (Fig. 4c, Supplementary Tables 9–12).

### Cancer and Alzheimer's genes distinguish foetal samples

For our final comparison, we investigated differences in adult and foetal samples based on donor metadata. All samples that were listed from a pre-birth donor were labelled as foetal samples; all samples labelled adult samples either came exclusively from adult donors (over 18 years old), or came partially from adult donors with no age information for other donors.

**Figure 5:**
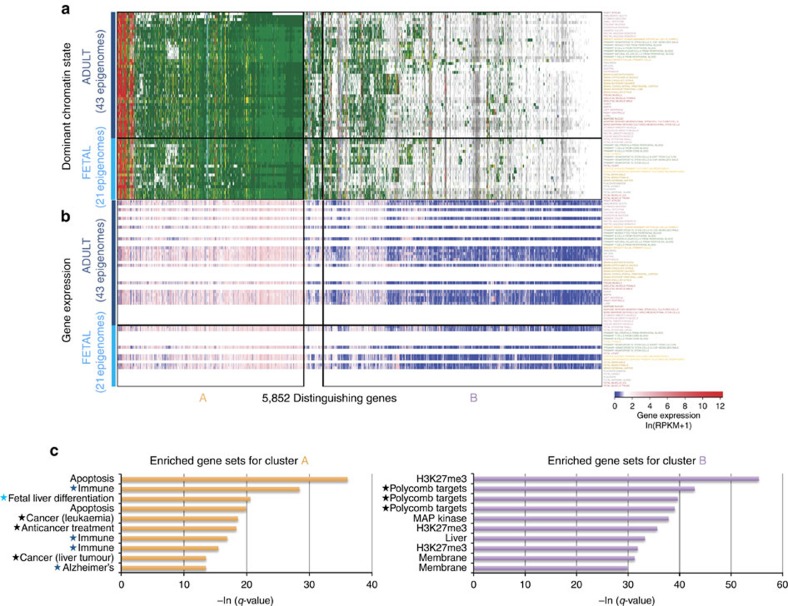
Cancer genes, Alzheimer's genes, and Polycomb targets distinguish adult and foetal samples. (**a**) The most abundant state of distinguishing genes for adult and foetal epigenomes is largely unchanged between the two groups, with the most popular states being active promoters, transcriptional, repressed or quiescent regions. (**b**) Gene expression profiles confirm similar levels of expression between the adult and foetal epigenomes at identified genes. (**c**) Genes in cluster A are enriched for age-related genes (notated by stars) relating to foetal cell differentiation, cancer, and Alzheimer's disease, while genes in cluster B are enriched for Polycomb targets, which have been shown to exhibit different behaviour in foetal and adult cells.

We found 7,472 significant epigenomic features distinguishing adult and foetal samples, spanning 5,852 unique genes. Visualization of the most abundant chromatin state of each gene in each epigenome (Fig. 5a) revealed that most significant genes had the same most abundant state in both adult and foetal epigenomes, suggesting more subtle underlying epigenomic changes at these genes. Specifically, although the most abundant chromatin state was usually transcribed or quiescent states, the underlying changing chromatin state spans all 15 chromatin states (Supplementary Fig. 5a). Specifically, many genes that were mostly quiescent or repressed were associated with changes in transcribed (Tx) and flanking active promoter (TssAFlnk) states, while genes that were mostly transcribed in all epigenomes exhibited changes in zinc finger (ZNF), quiescent (Quies) and weakly Polycomb repressed (ReprPCWk) state annotations (Supplementary Fig. 5b). Transcription was also similar between adult and foetal samples at most sampled genes (Fig. 5b), with only 15% of all distinguishing genes differentially expressed (887/5,798 distinguishing genes with expression data).

Overall, gene set enrichments for the 5,852 identified genes resulted in wide-ranging biological pathways, including gene sets related to liver, Polycomb targets and cytokines (Supplementary Table 13). However, from the visualization of the most abundant chromatin states (Fig. 5a), we identified two gene subgroups with distinctive epigenomic signatures and cohesive corresponding enrichments (Fig. 5c, Supplementary Tables 14–15). Cluster A genes are enriched for genes related to tumours and anticancer treatment response, as well as genes related to apoptosis; this result is supported by previous work that has shown that tumours have expression profiles similar to early developmental tissues^48^. Furthermore, cluster A is also enriched for genes related to differentiation of foetal liver cells, as well as genes related to immune response and Alzheimer's disease; this is particularly relevant given the increasingly recognized role of immune processes in Alzheimer's disease^49^ and that proteins known to affect foetal development also play a protective role for Alzheimer's disease^50^,^51^,^52^. On the other hand, genes in cluster B are enriched for membrane genes and Polycomb targets, which is relevant given the evidence that Polycomb proteins distinguish foetal and adult haematopoietic stem cells^53^,^54^. Taken together, this validates the ability of ChromDiff to identify relevant gene sets and pathways, despite a lack of change in expression data.

### Identified genes are enriched for differential expression

**Figure 6:**
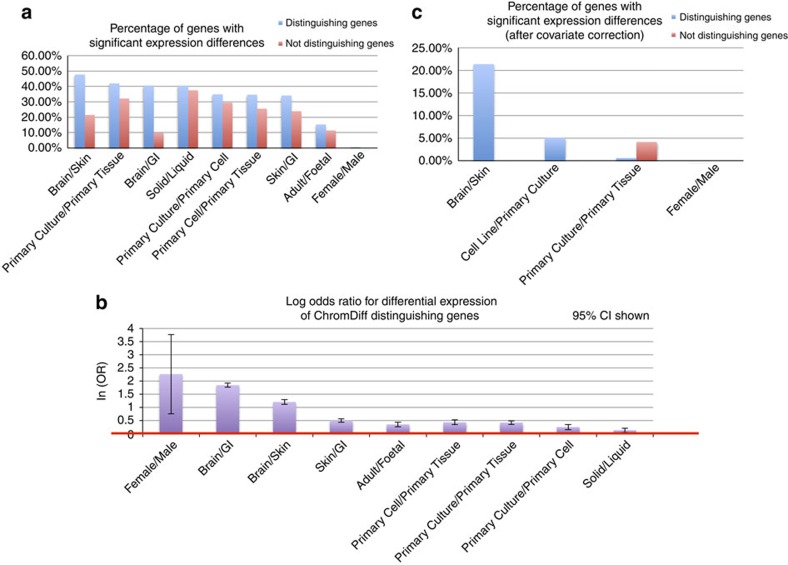
Epigenomically distinguishing genes are enriched for differential expression. By analysing expression of genes that our method identifies as being part of distinguishing gene and chromatin state combinations, we find that our method both recaptures differential gene expression and identifies distinguishing epigenetic context not captured by differential gene expression. (We performed this analysis on the 12 comparisons that produced distinguishing epigenomic features.) (**a**) Overall, identified genes are more differentially expressed than non-distinguishing genes, although <50% of the genes identified are significantly differently expressed. (The three comparisons that resulted in no differentially expressed genes are excluded.) (**b**) In every comparison of our nine remaining comparisons, our identified genes were significantly enriched for differentially expressed genes overall (as designated by asterisks), based on calculation of the log odds ratio and the corresponding 95% confidence intervals, as shown by the error bars. (*P*<0.003 in all cases, two-sided *z*-test) (**c**). After correcting for covariates in expression data, ChromDiff identifies all of the differentially expressed genes in two of the remaining four cases. (The eight comparisons that yielded no significant expression differences are excluded.)

The genes identified from our comparisons often exhibited different expression levels between the groups compared. To quantify this, for each of the 12 comparisons with distinguishing features (Supplementary Table 1), we calculated how many of our identified genes had differential gene expression between the 2 groups of the comparison (Fig. 6a) (see Methods). Three comparisons that revealed epigenomic differences did not have any differentially expressed genes: Brain/ESC, Cell Line/Primary Culture and ESC/GI. Furthermore, in the nine cases with differentially expressed genes, the epigenomically distinguishing genes included proportionally more differentially expressed genes than the non-distinguishing genes, with log odds ratios ranging from 0.13–2.26 and 95% confidence intervals as shown (Fig. 6b). In all nine cases, the increased proportion of differentially expressed genes is found to be significant, with the null hypothesis of ln(OR)=0, or equivalently odds ratio (OR)=1, falling outside the 95% confidence interval.

To more precisely identify genes directly associated with each biological feature, we further used linear regression to correct the reads per kilobase per million (RPKM) values for the same covariate factors previously used on the epigenomic features (production centre, sex of donor, sample state and sample type). After covariate correction, only four comparisons had any genes that were found to be differentially expressed: Brain/Skin, Cell Line/Primary Culture, Primary Culture/Primary Tissue and Female/Male. Surprisingly, all of the differentially expressed genes found in the Brain/Skin and Cell Line/Primary Culture comparisons were also identified by ChromDiff as distinguishing genes (Fig. 6c). On the other hand, many of the differentially expressed genes found in the Primary Culture/Primary Tissue and Female/Male comparison were not identified based on epigenomic changes, suggesting that expression and epigenomic comparative analyses are complementary methodologies.

Last, due to the fact that we had more samples with chromatin state data than gene expression data, we also applied ChromDiff to the original 17 comparisons while excluding any epigenomes without gene expression data. Due to the reduced power, in this case, only four biological comparisons yielded epigenomically distinguishing features and genes (Supplementary Table 16). However, for three of those biological comparisons, no differentially expressed genes were found after correcting for covariates, while the fourth comparison revealed that about 42% of the differentially expressed genes were identified by ChromDiff (Fig. 6d).

Overall, we have strong evidence that comparative chromatin state and differential gene expression methodologies are complementary approaches for comparative analysis. Whether we use covariate correction or limit ourselves to epigenomes with expression data, the main result is the same: the genes with differential expression are always a minority of the entire set of ChromDiff-identified genes, implying that many genes with epigenomic differences are not differentially expressed, and would be missed by differential gene expression analysis. While differential expression analysis has proven to be and will continue to be extremely useful, the chromatin state comparison provided by ChromDiff provides another lens through which comparative analysis can be viewed, and using these tools in combination improves the power of the overall analysis.

### ChromDiff outperforms other method for epigenomic comparison

To our knowledge, only one previous method exists to address comparison of epigenomic groups, and it utilizes PCA analysis on the differences between the means of the groups^40^. However, this approach presents a number of limitations. First, this approach innately ties the identification of combinatorial histone mark patterns to PCA analysis. Meanwhile, many segmentation methods have proven the usefulness of different machine learning approaches to identify combinatorial chromatin states, such as HMMs^21^,^23^ and Bayesian networks^22^. Since ChromDiff is compatible with any segmentation, it enables the use of a variety of existing and future methodologies. Second, public data resources such as Roadmap Epigenomics and ENCODE empower researchers everywhere to make discoveries through their analyses; however, these resources also necessitate less standardized data, as the data is often generated from a variety of labs, individuals and samples. ChromDiff corrects for covariate factors based on the metadata that the user provides, and in doing so it is uniquely positioned to perform comparative epigenomic analysis from these resource data sets. In contrast, PCA is designed for use with replicates, which limits the type and number of biological comparisons that can be performed from any given data set. Last, PCA is sensitive to relative scaling of values, while our rank-based statistical tests produce the same results regardless of scaled values.

To validate the expected improvement that ChromDiff provides for this analysis, we applied the differential PCA method^40^ to the Roadmap Epigenomics data for the same five histone marks used by ChromHMM: H3K4me3, H3K4me1, H3K36me3, H3K9me3 and H3K27me3 (ref. 41). dPCA reports whether any differential principal components were found, based on a cutoff of a signal-to-noise ratio of 5, since accuracy suffers substantially for components with a lower signal-to-noise ratio^40^. In three important comparisons, dPCA fails to recover any significant PCs, thereby generating no follow-up regions: specifically, dPCA found no genes with epigenomic differences for comparisons based on sex (Female/Male), developmental age (Adult/Foetal), and type (Cell Line/Primary Culture) (Supplementary Table 17). These results indicate that dPCA is unable to identify differences for two biological properties (sex and developmental age); furthermore, epigenomic sex differences due to X chromosome inactivation is one of the most studied and well-understood examples of epigenomic state differences, and as such it represents a ‘gold standard' that dPCA is unable to reproduce.

While dPCA is able to identify epigenomic differences for some comparisons when ChromDiff is not (Supplementary Table 17), the genes that dPCA identifies are less specific to the biological comparison, likely due to lack of covariate correction. For example, we noted that the gene set *lastowska neuroblastoma copy number dn* was in the top 2 enriched gene sets for every comparison (14 of 14) that produced results, and *chen liver metabolism qtl cis* was the other gene set in the top 2 for over half of the comparisons (8 of 14).

**Figure 7:**
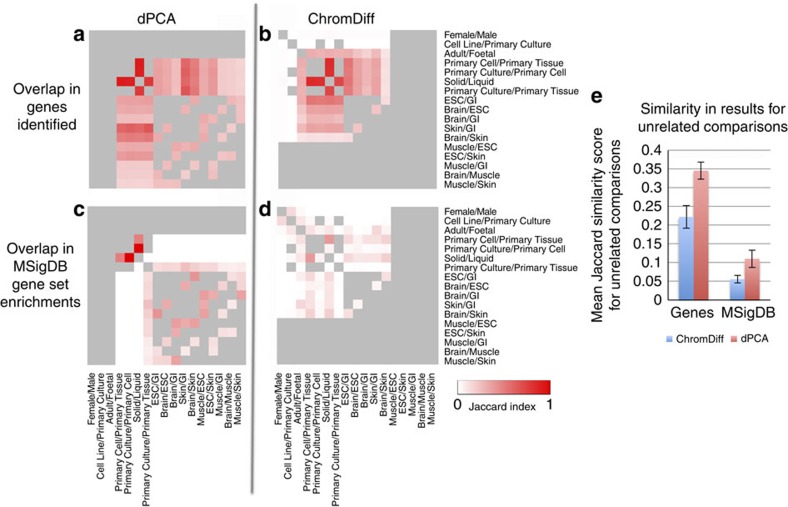
ChromDiff identifies more specific results than dPCA. After filtering out pairs of comparisons with shared epigenomic groups, we find that (**a**). dPCA's gene results are less specific than (**b**). ChromDiff's gene results for unrelated comparisons. Similarly, (**c**). dPCA's gene set enrichments are less specific than (**d**). ChromDiff's gene set enrichments for unrelated comparisons. (**e**) We quantify this result by confirming that dPCA's results have higher mean similarity scores for unrelated comparisons than ChromDiff does, with bars displaying s.e. of the sample mean. These scores were calculated from the 53 and 58 pairs of unrelated comparisons for ChromDiff and dPCA results, respectively, as shown in **a**–**d**.

To quantify this lack of specificity from dPCA, we calculated the Jaccard similarity score of the lists of distinguishing genes for pairs of comparisons, and as expected, we see higher similarity scores for the dPCA results than the ChromDiff results (Supplementary Fig. 6a,b). Even more strikingly, we see very high similarity between the enriched MSigDB gene sets for the genes identified by dPCA (Supplementary Fig. 6c), while ChromDiff returns gene set enrichments specific to that comparison (Supplementary Fig. 6d). Since some of these comparisons also share epigenomic groups (for example, the same brain epigenomes are used for Brain/ESC and Brain/GI), we filter out similarity scores for pairs of comparisons with overlapping groups (Fig. 7a–d). After filtering, we again find that dPCA has a higher average similarity score among unrelated comparisons than ChromDiff for both gene and MSigDB results (Fig. 7e).

These results show that for the Roadmap Epigenomics and ENCODE data sets, ChromDiff is more powerful than dPCA: ChromDiff can identify genes showing important epigenomic changes even when dPCA does not have enough power, and ChromDiff also identifies more specific and relevant gene sets than dPCA, likely due to its ability to correct for covariates.

Furthermore, ChromDiff also provides the ability to identify gene clusters, as well as groups of chromatin states that are acting through coordinated changes on the same regions. In this way, ChromDiff can further our understanding of how chromatin states complement and coordinate with one another.

With these results, we are confident that ChromDiff is a valuable contribution to the field of comparative epigenomics that can further our understanding of the relationship between chromatin states, genomic regions and biological features.

## Discussion

Overall, our method, ChromDiff, compares epigenomic states between groups of epigenomes to highlight chromatin state changes at genes with relevant functions. It accomplishes this by generating an information-theoretic encoding of each epigenome and isolating differences corresponding to a single biological attribute through covariate correction. Applications of our method reveal that different chromatin states and genes play important distinguishing roles in different comparisons. We further find an overall enrichment for differentially expressed genes in our identified gene sets, and in some cases, ChromDiff even identifies all differentially expressed genes based solely on the epigenomic data. We validate our methodology by showing that shuffled simulations almost always yield no epigenomic differences and that our approach outperforms the only previously existing method for group-wise epigenomic comparative analysis, particularly in terms of the specificity of the results. Due to these findings, we believe that our method is a powerful and innovative tool that will only increase in its ability to elucidate biological differences as more epigenomes become available.

The field of systems biology seeks to uncover the dynamics of gene regulatory processes in diverse biological functions including differentiation and disease. To date, analyses have focused primarily on differential gene expression analysis and epigenomic comparisons limited to histone mark signals. By comparing chromatin state annotations here, our results suggest that a rich set of molecular features distinguish gene activity across different biological parameters. Thus, we believe that our comparative analysis of chromatin states provides value by elucidating how the chromatin states associated with each gene vary across biological parameters and conditions. Previous methods for comparative epigenomic analysis have already been fruitful, but ChromDiff is ideally suited for analysis of resource data sets, due to its ability to control for external covariates and perform multiple group-wise comparisons using different attributes on the same data set. Therefore, as more epigenomes become available in less controlled settings, ChromDiff will continue to be a valuable tool and pipeline for research. Furthermore, using chromatin states rather than the raw underlying signal allows for an abstraction based on segmentation that can be used with varying underlying methodologies, and it also provides an additional lens through which the interplay of gene expression, epigenomic context and biological attributes and pathways can be examined.

The methods developed here address the question of set-to-set comparisons and leverage the multiple epigenomes in each set for the statistical tests performed. However, many other modelling frameworks utilizing chromatin states are possible, and ChromDiff opens the door to other potential statistical, machine learning and information-theoretic methodologies based on chromatin state segmentations. These future methods may also reveal new biological results and insights complementary to gene expression analysis and to the probabilistic, information-theoretic and statistical approach taken here.

Beyond the comparisons carried out here across sex, tissue type, suspension state and biological age, our method provides a model for diverse epigenomic comparisons. Our set-based analysis can be applied to compare epigenomes from groups of individuals, such as case–control disease studies, comparisons of cancer samples and normal controls from matched cell types, and longitudinal comparisons of matched individuals across treatments or interventions, to cite a few examples. ChromDiff can be easily expanded to include both distal and proximal intergenic regulatory regions, which has little effect on the distinguishing genes recovered when including promoter regions (Supplementary Fig. 7), but has a larger effect with much longer upstream regions that dominate the gene body length (Supplementary Fig. 8). In each of these cases, the stringent statistical approach that ChromDiff combines with covariate correction for external factors can be used to recognize chromatin states and clusters of relevant regions across groups of samples.

There is still much to be learned about the roles of epigenomic modifications in cellular activity, and much debate surrounds whether epigenomic features contribute as drivers of regulatory processes or simply as a consequence of transcription factor binding. Though our computational method cannot distinguish correlation from causation directly, our method and our results provide an important set of biological examples of highly statistically significant and recurrent epigenomic differences validated across large groups of epigenomes, by pointing out what regions, chromatin states and biological pathways distinguish the groups studied here. Moreover, the specific regions uncovered can guide future directed experimental studies to disentangle the relationship between the epigenomic changes and gene expression. Further, the individual genes and gene clusters revealed can be useful as priors for interpreting gene expression changes, enrichment analysis of disease-associated mutations from genome-wide association studies, target regions for regulatory motif analysis and, more generally, as priors for diverse applications seeking to mine common biological processes. As such, we believe our work will have important and diverse applications for understanding epigenomic variation more broadly.

## Methods

To summarize the epigenomic state of each cell type, we use the chromatin state annotations from ChromHMM^21^, as described in the integrative Roadmap Epigenomics paper^41^. To narrow our areas-of-interest and provide increased interpretability, this application of our method focuses on chromatin state annotations at gene bodies, but the method could generally be applied to any genomic regions.

### Chromatin state annotations

ChromDiff is applicable to any chromatin state annotation that is given. In our case, we have used the chromatin state annotations associated with the 15-state model from the Roadmap Epigenomics project based on the 5 core histone marks H3K4me3, H3K4me1, H3K36me3, H3K9me3 and H3K27me3 (ref. 41), including the annotations for epigenomic data from ENCODE^10^.

### Information-theoretic representation of raw feature values

Each feature is a combination of a gene and chromatin state: for gene *X* and chromatin state *Y*, we calculate the probability of gene *X*'s assignment to chromatin state *Y* in each epigenome, based on genomic per cent coverage of the maximum posterior probability chromatin state annotation^41^. Specifically, the raw feature value for feature_*X,Y*_ in epigenome *Z* is calculated as follows:


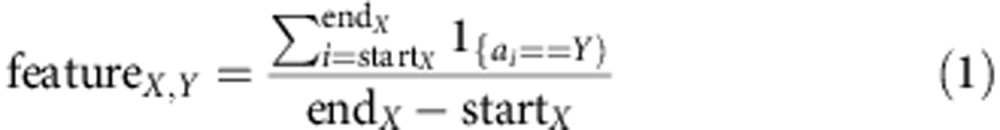


where start_*X*_ and end_*X*_, are the basepair locations of gene start and gene end of gene *X*, and *a*_*i*_ indicates the chromatin state annotation for epigenome Z at basepair *i*. (This equation implies usage for the applicable chromosome for gene *X*.)

### Covariate properties and traits

We corrected for four covariate property factors: (1) sex of the sample donor, (2) laboratory that processed the sample, (3) sample type and (4) whether the sample was a solid or liquid sample.

More specifically, four Roadmap Epigenome Mapping Centers (REMCs) contributed to the data: the Broad Institute (BI), the University of California, San Diego (UCSD), the University of California, San Francisco and the University of British Columbia (UCSF-UBC), and the University of Washington (UW). This resulted in four explanatory variables, with one for each lab. For any epigenome that was completely generated at a single lab, that lab's explanatory variable was given a value at 1, while the other labs were given 0. When multiple labs contributed to an epigenome, the corresponding lab covariate factor was calculated as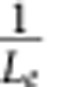, where *L*_*e*_ is the number of labs that contributed to epigenome *e*. Similarly, for sex if there were male and female donors for a given epigenome, a value of 1/2 was given for the female and male covariate values; otherwise, the variable for the donor's sex was given a 1 and the other sex variable was a 0. For sample types, the value for a covariate factor was 1 for the correct sample type and 0 for the other types, since each epigenome was annotated as one of the possible five possible sample types: cell line, derived cell line, cancer cell line, primary cell and primary tissue. For the sample state covariate factors, each sample was either annotated as solid, liquid or neither, which was translated into three corresponding explanatory variables with values of 1 for the correct annotation and 0 for the others^55^.

### Covariate correction of ChromDiff feature values

For each feature and each comparison, we fitted a logistic regression model to our raw feature values across all the epigenomes, excluding any covariate factors that were explicitly being tested by the comparison. As our raw feature values were bounded as fractional values between 0 and 1, logistic regression allowed for appropriate correction. Specifically, we used the generalized linear model (glm) function available in the stats package in *R*^56^. As defined below, we used the deviance residuals from our fitted logistic model as our corrected feature values.

Formally, if we are comparing traits *A* and *B* of property *C*, then we have *N*_*c*_ explanatory variables for our model, where *N*_*c*_ is the number of explanatory variables after excluding any that correspond to property *C*. As in standard logistic regression, we are modelling the 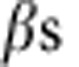 in the following formula:


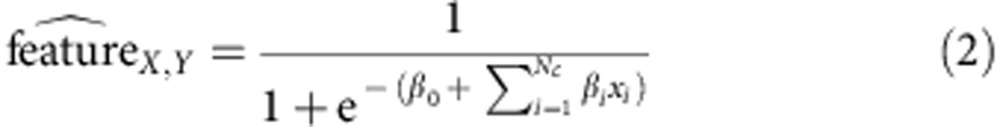


such that *x*_*i*_ corresponds to the value of the *i*^th^ explanatory variable for feature_*X,Y*_ for which we are adjusting.

Therefore, the corrected feature values take on the value of the deviance residual, which is:


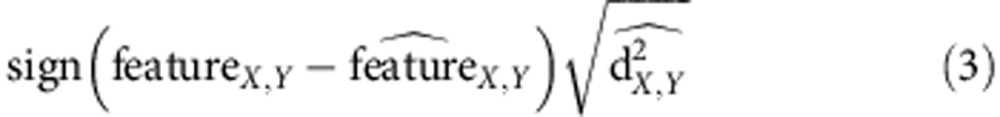


where:


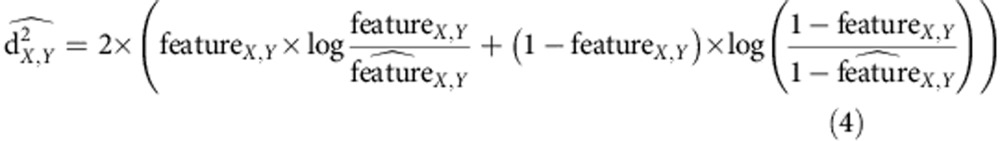


A final detail to note is that logistic regression requires the conversion of fractional values to ‘successes' and ‘failures', so we convert the feature_*X*,*Y*_ fractions into feature_*X*,*Y*_ × *N* successes and (1−feature_*X*,*Y*_) × *N* failures, where *N* is the length of gene X, or end_*X*_−start_*X*_.

As described in `Covariate properties and traits', we converted the categorical covariate factors into ‘continuous' explanatory variables; if there were *c* categories for a certain factor, this resulted in *c* explanatory variables for that factor by converting Boolean variables into binary values. For samples that were mixtures of *n* categories, each corresponding explanatory variable was given a value of *1/n*.

### Gene annotations

We used all protein-coding genes with corresponding gene ids and positions as given in GENCODE v10 (ref. 57) for compatibility with the Roadmap Epigenomics Consortium^41^, with the exception of genes encoded on chromosome Y, which we omitted. Gene symbols for gene set enrichment calculations were also taken from the GENCODE annotations.

### Statistical test

ChromDiff currently supports three statistical tests: the two-sided *t*-test, the *F*-tests and the Mann–Whitney–Wilcoxon test. Any of these tests can be used to identify features that show statistically different feature values across the two groups. In the presented results, we used only the Mann–Whitney–Wilcoxon test.

### Multiple hypothesis correction

ChromDiff supports Bonferroni, Benjamini–Hochberg or Benjamini–Yekuteli multiple hypothesis correction^56^,^58^,^59^ on *P* values based on the number of features tested for each comparison. In this analysis, we used Benjamini–Hochberg false discovery rate (FDR) correction. Features that had a corrected *P* value <0.05 after correction were considered significant.

Though our features are slightly dependent on one another due to the connection between each gene and its 15 features, Benjamini–Hochberg FDR correction is always valid for dependent tests that uphold positive regression dependency in a subset, and Benjamini–Hochberg also performs well in many practical cases and simulations^60^,^61^,^62^,^63^,^64^. For completeness, we also compared the number of distinguishing features found with the more conservative Benjamini–Yekuteli procedure^59^, which controls the FDR under any dependency or distribution environment, and we found that most of our biological comparisons still result in significant chromatin state differences (Supplementary Fig. 9).

### Gene set enrichment calculations

Once we identified genes that are associated with at least one significant distinguishing feature, we calculated hypergeometric^65^ *P* values for gene sets from MSigDB^66^, effectively using the Fisher's exact test with Storey's FDR *q*-value correction^67^,^68^. The databases used from MSigDB were C2 (curated) and C5 (Gene Ontology) gene sets, downloaded based on gene symbols. We used the gene symbols associated with each gene, as provided by GENCODE gene IDs. This analysis was performed on all the distinguishing genes identified in a comparison, as well as the clusters of sampled genes identified, as described in `Sampling distinguishing genes' and `Gene cluster identification.'

### Expression data

RNA-Seq data from the Roadmap Epigenomics and ENCODE projects^10^,^41^ were used, when available. Specifically, the per-gene RPKM values provided by Roadmap Epigenomics^41^ for ENSEMBL-defined protein-coding genes were used.

### Sampling distinguishing features

For visualizations, we sampled down to 10,000 distinguishing features when more features than this were identified. The sampled features and their associated genes were then used for all heatmaps, such as gene and chromatin state combination plots, most abundant (dominant) chromatin state plots and gene expression difference plots. To sample the 10,000 distinguishing features that would be most informative, we prioritized genes that were associated with the greatest number of significant distinguishing features, breaking ties based on the *P* value of the most significant associated feature. Then, all features associated with the prioritized genes were chosen, until 10,000 features were reached.

### Sampling distinguishing genes

All genes corresponding to sampled distinguishing features were retained.

### Gene cluster identification

All clustering was based on hierarchical clustering using the complete linkage method based on Euclidean distances, as implemented by hclust in the R stats package^56^. After obtaining the dendrogram for the hierarchical clustering, we manually identified a cutoff for each comparison while taking into account cluster homogeneity and size. The resulting clusters were annotated on the heatmaps if they included at least 5% of all elements clustered. We also performed calculations for enriched gene sets and differentially expressed genes on these annotated clusters, as described in ‘gene set enrichment calculations' and ‘significant expression differences of gene groups'.

### Ordering of rows or columns

When ordering rows or columns, the dendrogram was generated based on the matrix values, which was then ordered by the means of the vectors.

### Ordering of feature combination plots

When visualizing features as chromatin state and gene combinations, we set each cell value of the matrix to be the corresponding chromatin state number between 1 and 15 (based on the chromatin state order shown in Fig. 1a) if that gene (column) and chromatin state (row) combination was found to be a significant feature. Otherwise, the cell value was set to not applicable (NA). The colour scale for the heatmap was set so that each chromatin state number would map to its colour (as shown in Fig. 1a), with rows and columns ordered as described in `Ordering of rows and columns'.

### Dominant chromatin state heatmaps

For each comparison, these heatmaps visualized the sampled distinguishing genes as columns and relevant epigenomes as rows. Each cell shows the corresponding colour for which chromatin state was most present for that gene in that epigenome. Colours for chromatin state were taken from the ChromHMM state colouring as given by the integrative Roadmap Epigenomics project (shown in Fig. 1a). Cell type and gene orderings were calculated as described in 'Ordering of dominant chromatin state heatmaps'.

### Ordering of dominant chromatin state heatmaps

The columns were ordered based on the column means according to the dendrogram for hierarchical clustering described in ‘gene cluster identification'. The rows were ordered separately for the two biological groupings. First, for each group, we identified the dendrogram for the corrected feature values via hierarchical clustering of hclust^56^ and ordered the dendrogram based on the row means. Then, we simply concatenated the orderings for the two groups.

### Gene expression heatmaps

Row (cell type) and column (gene) orderings were copied from the dominant chromatin state heatmaps, as described in ‘ordering of dominant chromatin state heatmaps'. The colour of each cell represents the corresponding 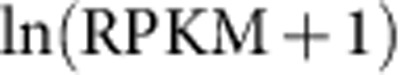 value for each gene and epigenome combination. The colour scale sets the minimum expression value to be red, the median to be white, and the maximum to be blue. Any epigenomes that had no expression data were plotted as white rows, for ease of comparison with other heatmaps.

### Significant expression differences of gene groups

*P* values were calculated using the two-sided Mann–Whitney-Wilcoxon test on all expression values of relevant genes in one group of epigenomes against the other group of epigenomes. This analysis was performed on all the distinguishing genes identified in a comparison, as well as the clusters of sampled genes identified, as described in 'Sampling distinguishing genes' and 'Gene cluster identification'.

### Proportion of genes with differential gene expression

For each ChromDiff-identified gene (a gene associated with a significant feature for a given comparison), a two-sided Mann–Whitney-Wilcoxon test was used on gene expression values between each grouping of epigenomes. Benjamini–Hochberg FDR correction^58^ was used on these *P* values, and genes with adjusted *P* values <0.05 were considered to have significant differential gene expression. This allowed us to calculate the per cent of significant genes that had differential gene expression. This analysis was repeated for all the genes that were not identified by ChromDiff, for comparative purposes and to calculate the log odds ratios (see ‘Odds ratio calculation').

### Odds ratio calculation

We calculated the log odds ratio, 95% confidence interval and corresponding *P* values to quantify the relationship between epigenomically distinguishing genes and differentially expressed genes^69^,^70^. Specifically, we can define *a* as the number of distinguishing genes with differential expression, *b* as the number of distinguishing genes without differential expression, *c* as the number of non-distinguishing genes with differential expression and *d* as the number of non-distinguishing genes without differential expression. The log odds ratio, or ln(OR), is therefore 
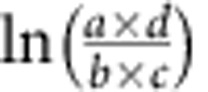
.

S.e. of the log odds ratio is calculated as
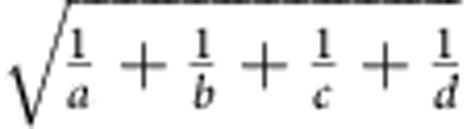
. The 95% confidence interval of the log odds ratio is defined as 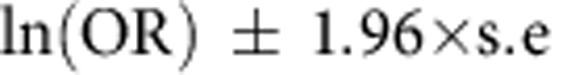. Using a *P* value threshold of 0.05, there is a significant relationship between the distinguishing genes and differentially expressed genes when the 95% confidence interval for the log odds ratio does not include 0.

Specifically, the *P* value can be calculated from the *z*-score, as 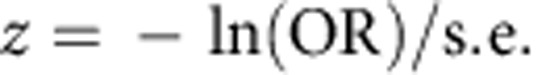, and the corresponding *P* value is e^−.717 × *z*−.416 × *z*^2^^.

### Covariate correction for gene expression

Covariate correction was performed in the same way as described above (see ‘Covariate correction for ChromDiff feature values the residuals were based on'), except that residuals were based on linear regression instead of logistic regression, due to the unbounded nature of RPKM values.

### Randomized comparisons

To confirm the biological relevance of our results, we performed 100 randomization tests for each biological comparison. During each randomization trial, we randomly shuffled the labels on the epigenomes we were testing, thereby retaining groups of matched size to the original comparison. Then we performed the same covariate correction and statistical testing as described above, and counted the number of significant distinguishing features found, if any.

Formally, let *X*_*A*_ and *X*_*B*_ be the sets of epigenomes that correspond, respectively, to the traits *A* and *B* of category *C*. (For example, *A*=Female, *B*=Male, *C*=sex of donor.) Then define 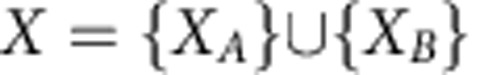. For each randomization trial 
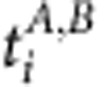
, for 1≤*i*≤100, randomly draw a new *X*_*A*_′ from *X* of size 
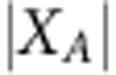
 with uniform probability (without replacement). Then define *X*_*B*_′=*X*−*X*_*A*_′, which means, by construction, that *X*_*B*_′ will be of size 
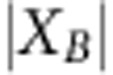
. Then, as described above, perform covariate correction for every category *C*′ such that *C*′≠*C*, and perform the statistical Mann–Whitney–Wilcoxon test to identify distinguishing features as usual.

We then summarized our findings as shown in Fig. 1e and Fig. 1f, by counting the fraction of all randomized trials that resulted in any significant distinguishing features, and contrasting this with the fraction of biological comparisons that resulted in found significant distinguishing features. Specifically, Fig. 1e depicts, in blue, the following fraction:


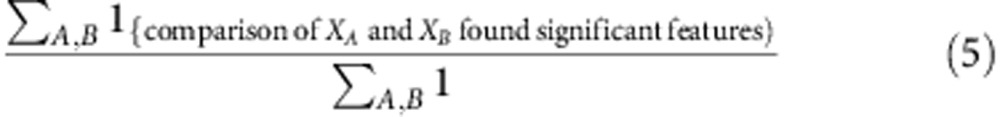


Specifically, Fig. 1f depicts, in blue, the following fraction:


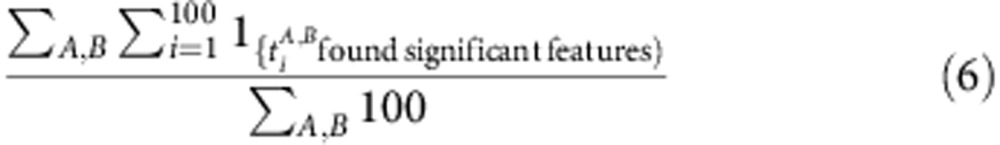


### Code

Code for ChromDiff, results presented here, instructions for usage, data used here and additional information can be found at http://compbio.mit.edu/ChromDiff. ChromDiff is freely available for download and usage under a GPL 3 license.

## Additional information

**How to cite this article:** Yen, A. & Kellis, M. Systematic chromatin state comparison of epigenomes associated with diverse properties including sex and tissue type. *Nat. Commun.* 6:7973 doi: 10.1038/ncomms8973 (2015).

## Supplementary Material

Supplementary Information Supplementary Figures 1-9 and Supplementary Tables 1-17
