# Supplementary material for: Systematic chromatin state comparison of epigenomes associated with diverse properties including sex and tissue type
Source: Nat Commun. 2015 Aug 18;6:7973. doi: 10.1038/ncomms8973 (PMC4557131; doi:10.1038/ncomms8973)
Supplement: Supplementary Information — Supplementary Figures 1-9 and Supplementary Tables 1-17 [file ncomms8973-s1.pdf]

Supplementary Figures

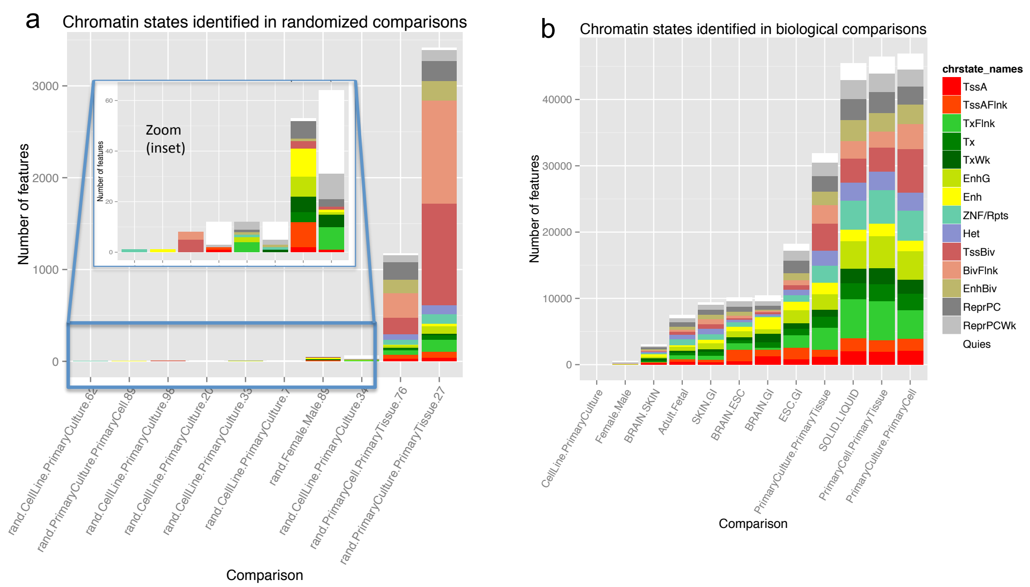

Supplementary Figure 1: A variety of chromatin states are identified in simulations and applications. a. In randomized comparisons, we find a wide variety of chromatin state distributions in the distinguishing features found, while in **b.** biological comparisons, we found that all the chromatin states were well represented.

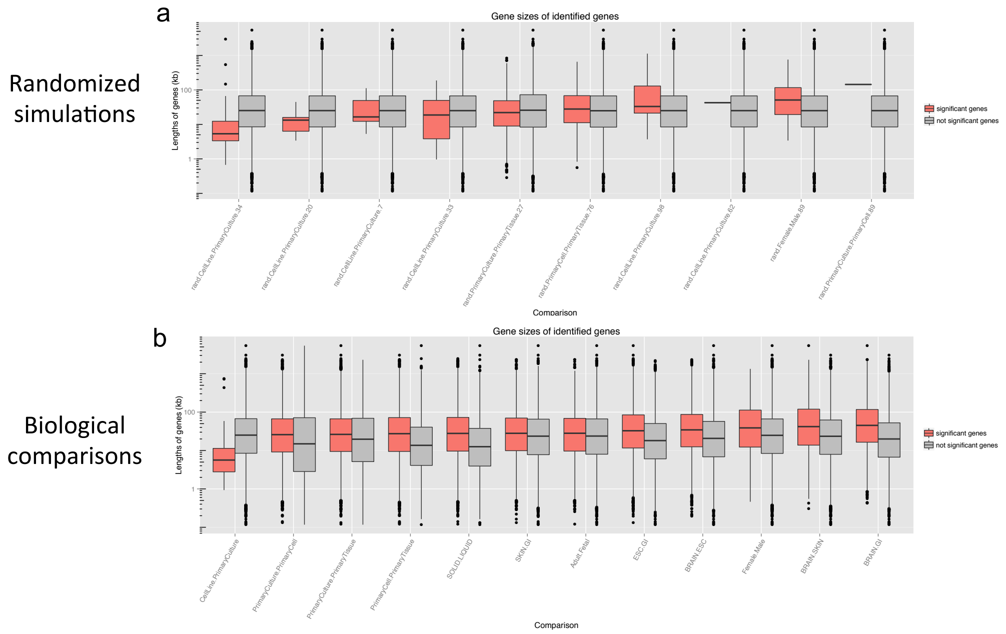

Supplementary Figure 2: Genes of various sizes are identified in randomized simulations, while longer genes are identified in biological comparisons. a. In randomized simulations, we identify genes with a variety of gene sizes, suggesting ChromDiff does not bias for certain gene sizes. **b.** In biological comparisons, the genes identified were often longer, suggesting that longer genes exhibit more epigenomic changes.

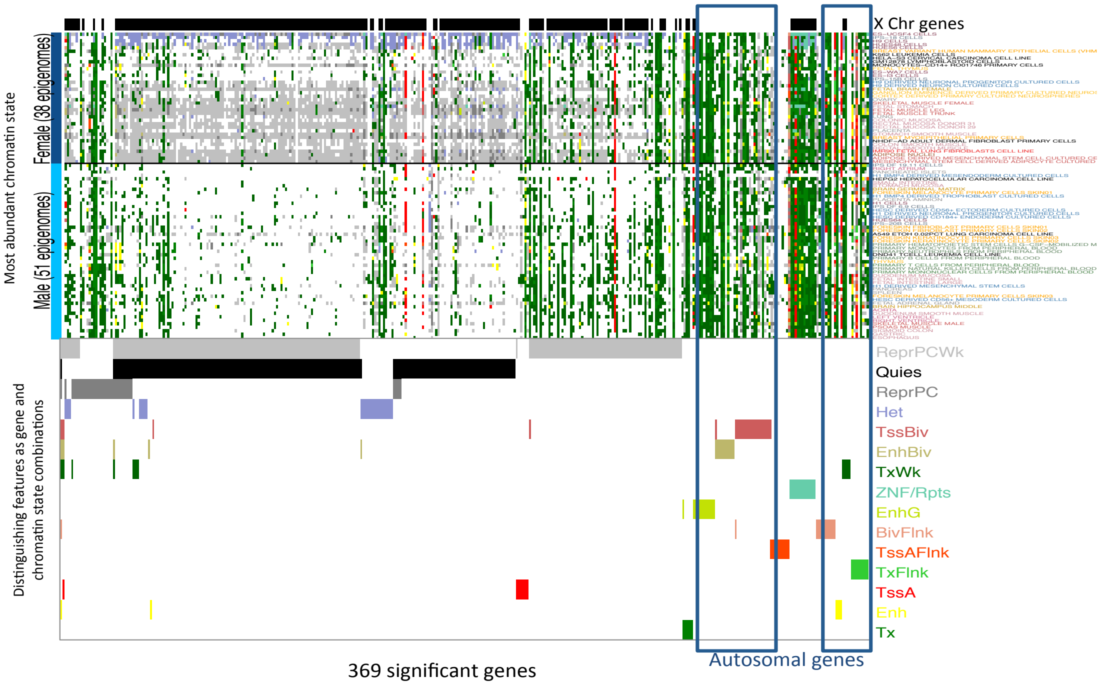

Supplementary Figure 3: Distinguishing autosomal genes are associated with changes in bivalent and enhancer regions. While X chromosome genes are largely associated with changes in quiescent and polycomb repressed regions, transcribed autosomal genes (highlighted in blue) are largely associated with bivalent and enhancer regions, as well as flanking transcribed (TxFlnk) regions.

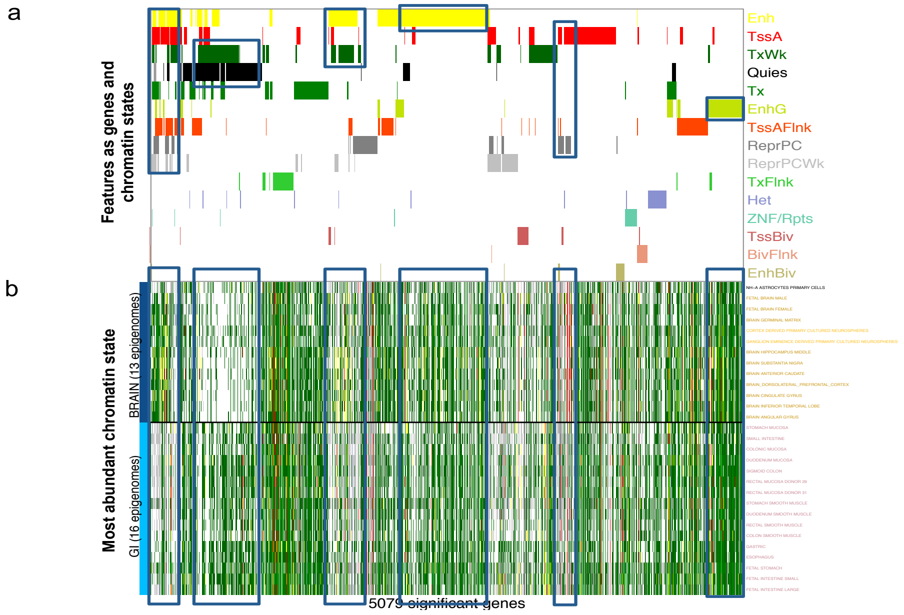

Supplementary Figure 4: Brain and gastrointestinal differences reveal changing chromatin state differences in gene clusters. a. Various chromatin states exhibit coordinated changes at corresponding gene clusters. Specifically, from left to right, the groups of chromatin states (highlighted in blue) that change at the same genes are a) enhancer and promoter regions (Enh/TssA/TssAFlnk), b) transcribed and quiescent (TxWk/Quies), c) enhancer and transcribed (Enh/TxWk), d) enhancer (Enh), e) promoter and repressed (TssA,/ReprPC), and f) genic enhancer (EnhG) regions. **b.** For groups a and c, gastrointestinal (GI) tissues are mostly quiescent, while group b genes are mostly transcribed in GI tissues. Group c and d genes are more often enhancer regions in brain samples, while group e genes are annotated as promoter states in both brain and GI samples.

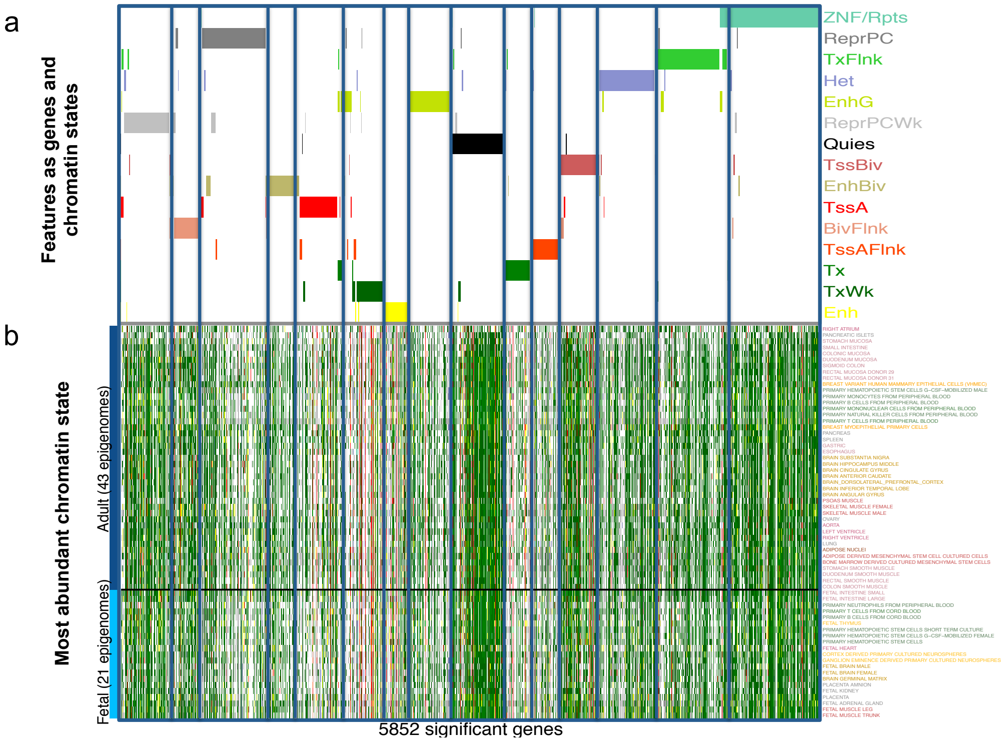

Supplementary Figure 5: Many genes exhibiting changes between adult and fetal samples are only associated with one chromatin state. a. Visualization of features as chromatin states and genes show that most genes are identified by changes due to only one chromatin state, rather than coordinated changes of multiple chromatin states. The most common chromatin states to differ between adult and fetal epigenomes were ZNF/Rpts, ReprPC, Quies, TxFlnk, Het, and ReprPCWk regions. **b.** Different patterns for the most abundant chromatin state can be seen for genes associated with different chromatin states; for example, genes with changes due to the Tx chromatin state are largely quiescent in both groups, while genes with changes due to ZNF/Rpts are mostly transcribed in both groups.

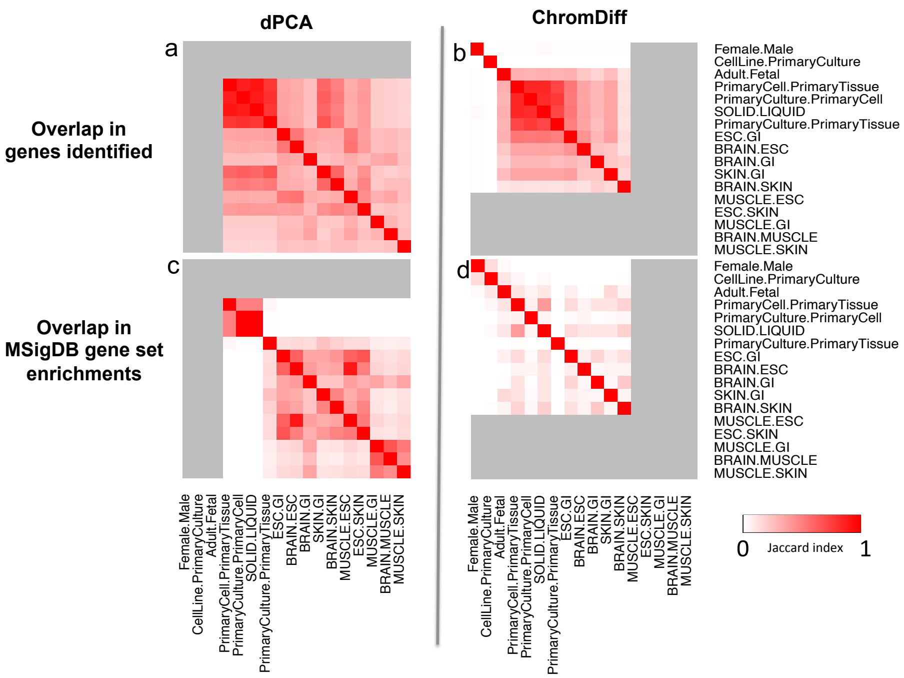

Supplementary Figure 6: ChromDiff outperforms dPCA in identification of comparison-specific genes a. dPCA re-discovers the same genes in many of its varying biological comparisons, while **b.** ChromDiff more frequently identifies different genes in different comparisons, based on the Jaccard index. Similarly, **c.** the enriched gene sets and pathways identified by dPCA are markedly similar for many of their comparisons, while **d.** ChromDiff achieves higher specificity that produces different, relevant gene set enrichments in different cases, based on the Jaccard similarity index.

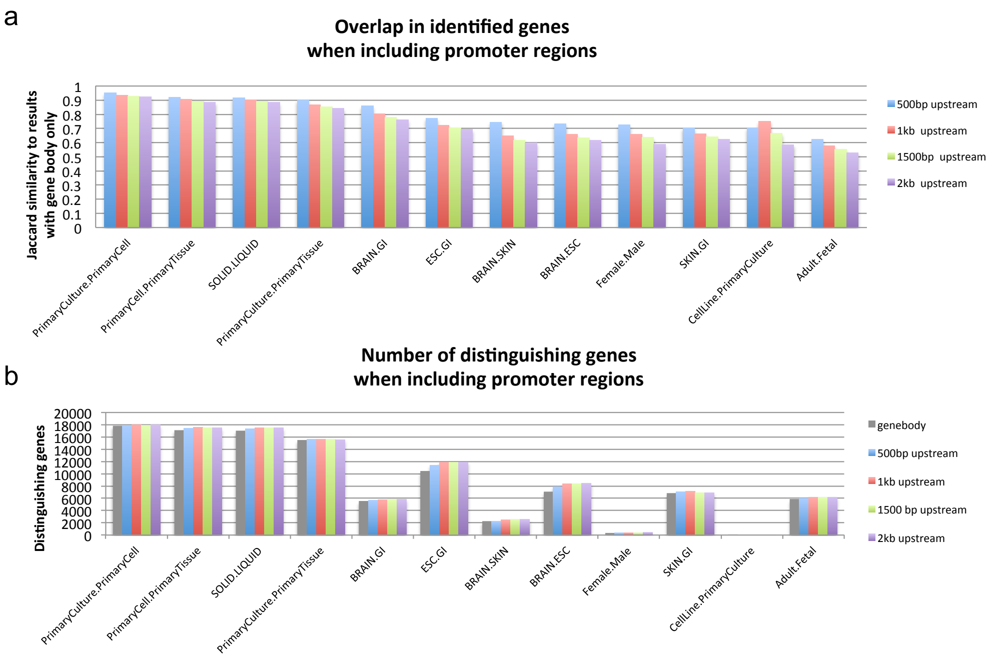

Supplementary Figure 7: ChromDiff is robust to adding promoter regions. When including upstream promoter regions with the gene body, we find very similar results, both in terms of **a.** the distinguishing genes identified and **b.** the power of ChromDiff to identify distinguishing genes.

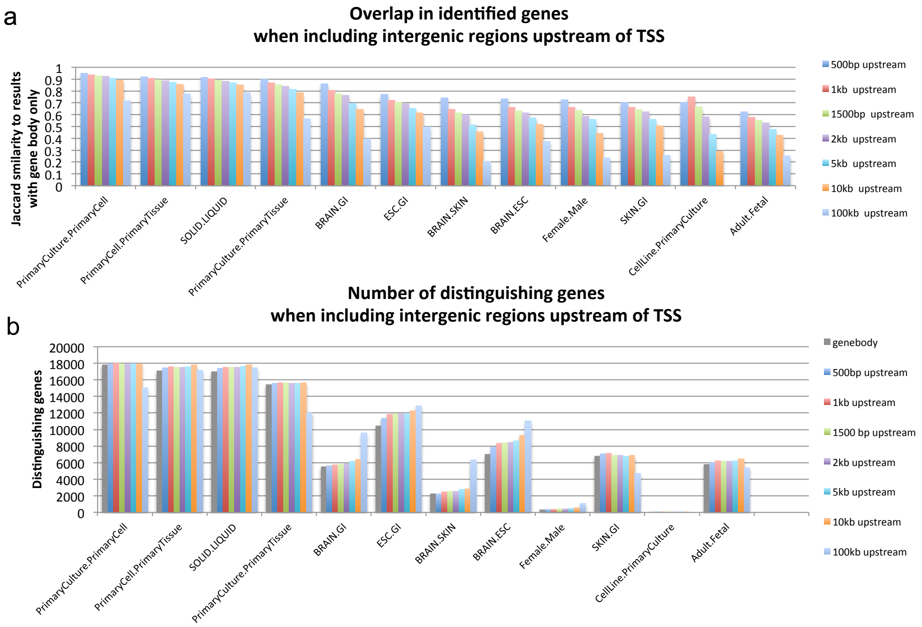

Supplementary Figure 8: ChromDiff identifies different results when including long intergenic regions. When including longer intergenic regions, we find **a.** different distinguishing genes and **b.** different power to identify regions compared to the gene body-centric approach.

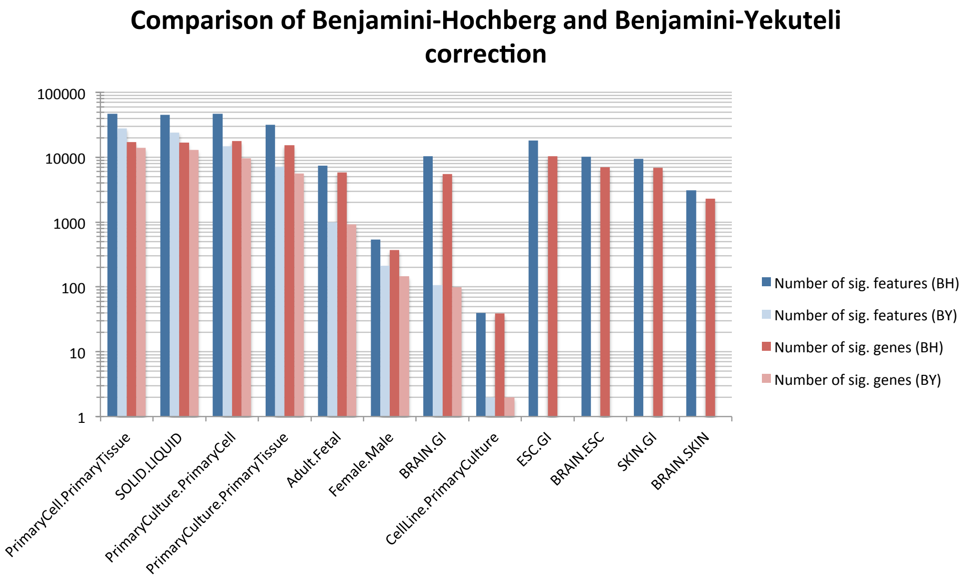

Supplementary Figure 9: Even with the conservative Benjamini-Yekuteli multiple hypothesis correction, we still identify significantly different features and genes in 7 of 12 cases. As Benjamini-Yekuteli multiple hypothesis correction is applicable for any distribution or dependency structure, we quantified the effect that it would have on our significant results. We found that in 7 of the 12 comparisons (and in all four comparisons presented in this paper), ChromDiff would still identify many significant features and genes using Benjamini-Yekuteli correction.

Supplementary Tables

Supplementary Table 1: Analysis of biological comparisons reveal differences in chromatin state

Group 1	Group 2	Property	Distinguishing features found	Height cutoff
CellLine	PrimaryCulture	type	TRUE	39
PrimaryCulture	PrimaryCell	type	TRUE	69
PrimaryCulture	PrimaryTissue	type	TRUE	83
PrimaryCell	PrimaryTissue	type	TRUE	83
Adult	Fetal	age	TRUE	80
Female	Male	sex	TRUE	80
BRAIN	MUSCLE	anatomy	FALSE	N/A
BRAIN	ESC	anatomy	TRUE	49
BRAIN	SKIN	anatomy	TRUE	44
MUSCLE	ESC	anatomy	FALSE	N/A
MUSCLE	SKIN	anatomy	FALSE	N/A
ESC	SKIN	anatomy	FALSE	N/A
BRAIN	GI	specialgi	TRUE	50
SKIN	GI	specialgi	TRUE	48
ESC	GI	specialgi	TRUE	48
MUSCLE	GI	specialgi	FALSE	N/A
SOLID	LIQUID	solid_liquid	TRUE	88

Of the 17 comparisons analyzed that spanned many groups and metadata properties, 12 comparisons identified differences in chromatin state between the two groups. For those cases, gene clusters were selected based on a manually chosen height cutoff for hierarchical clustering.

Supplementary Table 2: Enriched gene sets for cluster A of brain and gastrointestinal comparison

annotation	geneset	qval
cancer	NUYTTEN_EZH2_TARGETS_UP	7.73E-14
Alzheimer's	BLALOCK_ALZHEIMERS_DISEASE_UP	7.73E-14
parvin family of actin-binding protein (cytoskeleton, cell adhesion)	JOHNSTONE_PARVB_TARGETS_3_UP	1.11E-13
aggressive tumors	ONKEN_UVEAL_MELANOMA_UP	4.54E-12
apoptosis	GRAESSMANN_APOPTOSIS_BY_DOXORUBICIN_UP	4.54E-12
fibronectin response	MILI_PSEUDOPODIA_HAPTOTAXIS_DN	1.66E-11
tumor suppressor genes	LOPEZ_MBD_TARGETS	1.94E-11
TGF-beta signaling	KOINUMA_TARGETS_OF_SMAD2_OR_SMAD3	1.16E-10
cancer	REN_ALVEOLAR_RHABDOMYOSARCOMA_DN	1.50E-10

Comparison of brain and gastrointestinal samples show that distinguishing genes in cluster A are related to Alzheimer’s disease. (10 most strongly enriched gene sets shown.)

Supplementary Table 3: Enriched gene sets for cluster B of brain and gastrointestinal comparison

annotation	geneset	qval
cancer	RODRIGUES_THYROID_CARCINOMA_POORLY_DIFFERENTIATED_DN	1.54E-02

Comparison of brain and gastrointestinal samples show that distinguishing genes in cluster B are related to thyroid carcinoma. (Only one significantly enriched gene set found.)

Supplementary Table 4: Enriched gene sets for cluster C of brain and gastrointestinal comparison

annotation	geneset	qval
HCP, H3K27me3	MIKKELSEN_MCV6_HCP_WITH_H3K27ME3	5.65E-08
nervous system	GOBERT_OLIGODENDROCYTE_DIFFERENTIATION_DN	4.36E-04
nervous system	NERVOUS_SYSTEM_DEVELOPMENT	4.80E-04
nervous system	LEIN_NEURON_MARKERS	1.03E-03
H3K27me3	BENPORATH_ES_WITH_H3K27ME3	1.15E-03
HCP, H3K27me3	MIKKELSEN_MEF_HCP_WITH_H3K27ME3	1.90E-03
nervous, HCP	MEISSNER_NPC_HCP_WITH_H3K4ME2	2.48E-03
cancer	SMID_BREAST_CANCER_BASAL_UP	2.48E-03
nervous	MARTORIATI_MDM4_TARGETS_NEUROEPITHELIUM_DN	3.50E-03
nervous	LEE_NEURAL_CREST_STEM_CELL_DN	5.35E-03

Comparison of brain and gastrointestinal samples show that distinguishing genes in cluster C are related to the nervous system and H3K27me3 modifications. (10 most strongly enriched gene sets shown.)

Supplementary Table 5: Enriched gene sets for cluster D of brain and gastrointestinal comparison

annotation	geneset	qval
nervous	LEIN_OLIGODENDROCYTE_MARKERS	1.49E-03
psychiatric disorder	ASTON_MAJOR_DEPRESSIVE_DISORDER_DN	1.49E-03
brain	LU_AGING_BRAIN_UP	1.49E-03
HCP	MIKKELSEN_MEF_HCP_WITH_H3K27ME3	1.49E-03
cancer	BRUINS_UVC_RESPONSE_VIA_TP53_GROUP_A	1.49E-03
cell surface interactions	PID_INTEGRIN1_PATHWAY	4.93E-03
knockdown of proto-oncogene	YANG_BCL3_TARGETS_UP	4.93E-03
nervous	NERVOUS_SYSTEM_DEVELOPMENT	4.93E-03
brain, HCP	MEISSNER_BRAIN_HCP_WITH_H3K4ME3_AND_H3K27ME3	4.93E-03
nervous, HCP	MEISSNER_NPC_HCP_WITH_H3K4ME2	5.68E-03

Comparison of brain and gastrointestinal samples show that distinguishing genes in cluster D are related to psychiatric disorders, brain function, and the nervous system. (10 most strongly enriched gene sets shown.)

Supplementary Table 6: Enriched gene sets for cluster E of brain and gastrointestinal comparison

annotation	geneset	qval
gastric cancer	ONDER_CDH1_TARGETS_2_DN	2.04E-20
cancer drug sensitivity	COLDREN_GEFITINIB_RESISTANCE_DN	8.28E-20
cancer	CHARAFE_BREAST_CANCER_LUMINAL_VS_MESENCHYMAL_UP	2.70E-14
brain	MEISSNER_BRAIN_HCP_WITH_H3K4ME3_AND_H3K27ME3	3.02E-11
mammary stem cell	LIM_MAMMARY_STEM_CELL_DN	7.31E-09
cancer	CHARAFE_BREAST_CANCER_BASAL_VS_MESENCHYMAL_UP	5.28E-07
cancer	LIU_PROSTATE_CANCER_DN	5.28E-07
breast	MCBRYAN_PUBERTAL_BREAST_3_4WK_UP	6.31E-07
cancer	DELYS_THYROID_CANCER_UP	7.87E-07
cancer	DODD_NASOPHARYNGEAL_CARCINOMA_UP	8.18E-07

Comparison of brain and gastrointestinal samples show that distinguishing genes in cluster E are related to brain function and a variety of cancer types. (10 most strongly enriched gene sets shown.)

Supplementary Table 7: Enriched gene sets for cluster F of brain and gastrointestinal comparison

annotation	geneset	qval
H3K27me3	MIKKELSEN_MEF_HCP_WITH_H3K27ME3	8.83E-52
H3K27me3	BENPORATH_ES_WITH_H3K27ME3	1.58E-35
polycomb targets	BENPORATH_SUZ12_TARGETS	1.35E-28
H3K27me3	MIKKELSEN_MCV6_HCP_WITH_H3K27ME3	1.02E-26
polycomb targets	BENPORATH_EED_TARGETS	4.52E-26
nervous, H3K27me3	MEISSNER_NPC_HCP_WITH_H3K4ME2_AND_H3K27ME3	3.90E-22
membrane	PLASMA_MEMBRANE	7.82E-20
nervous, H3K27me3	MEISSNER_BRAIN_HCP_WITH_H3K4ME3_AND_H3K27ME3	1.34E-19
nervous	REACTOME_NEURONAL_SYSTEM	3.37E-19
membrane	PLASMA_MEMBRANE_PART	2.24E-18

Comparison of brain and gastrointestinal samples show that distinguishing genes in cluster F are related to polycomb targets, psychiatric disorders, brain function, and the nervous system. (10 most strongly enriched gene sets shown.)

Supplementary Table 8: Enriched gene sets for brain and gastrointestinal comparison

annotation	geneset	qval
brain, H3K27me3, high CPG density promoters (HCP)	MEISSNER_BRAIN_HCP_WITH_H3K4ME3_AND_H3K27ME3	1.97E-29
H3K27me3, HCP	MIKKELSEN_MEF_HCP_WITH_H3K27ME3	5.19E-26
CDH1 associated with gastric diseases	ONDER_CDH1_TARGETS_2_DN	3.64E-20
H3K27me3	BENPORATH_ES_WITH_H3K27ME3	1.34E-19
H3K27me3, HCP	MIKKELSEN_MCV6_HCP_WITH_H3K27ME3	2.70E-16
nervous, HCP	MEISSNER_NPC_HCP_WITH_H3K4ME2	3.79E-16
membrane	PLASMA_MEMBRANE	1.69E-15
cancer drug sensitivity	COLDREN_GEFITINIB_RESISTANCE_DN	3.38E-15
polycomb target	BENPORATH_SUZ12_TARGETS	6.72E-14
cellular processes	PEREZ_TP53_TARGETS	1.43E-13

Comparison of brain and gastrointestinal samples show that the total set of distinguishing genes are related to brain and nervous system function, as well as gastric diseases. (10 most strongly enriched gene sets shown.)

Supplementary Table 9: Enriched gene sets for cluster A from comparison of blood and non-blood samples

annotation	geneset	qval
leukemia (blood cancer)	MARTENS_BOUND_BY_PML_RARA_FUSION	7.62E-06
lymphoma (blood cancer)	YU_MYC_TARGETS_DN	4.70E-05
T cell differentiation	LEE_DIFFERENTIATING_T_LYMPHOCYTE	4.70E-05
macrophage (white blood cell)	CHEN_METABOLIC_SYNDROM_NETWORK	4.70E-05
immune response (hepatitis B viral clearance)	WIELAND_UP_BY_HBV_INFECTION	1.65E-04
lymphoma (blood cancer)	PASQUALUCCI_LYMPHOMA_BY_GC_STAGE_DN	4.65E-04
cancer	LINDGREN_BLADDER_CANCER_CLUSTER_2B	4.65E-04
cancer	WALLACE_PROSTATE_CANCER_RACE_UP	1.09E-03
leukemic progenitor cells (blood cancer)	TORCHIA_TARGETS_OF_EWSR1_FLI1_FUSION_DN	1.92E-03
tumor suppressor targets	SANSOM_APC_TARGETS_DN	6.01E-03

Comparison of blood and non-blood samples show that the total set of distinguishing genes in cluster A are related to leukemia, lymphoma, and immune response. (10 most strongly enriched gene sets shown.)

Supplementary Table 10: Enriched gene sets for cluster B from comparison of blood and non-blood samples

annotation	geneset	qval
macrophage (white blood cell)	FOSTER_TOLERANT_MACROPHAGE_DN	6.50E-08
macrophage-enriched metabolic network	CHEN_METABOLIC_SYNDROM_NETWORK	7.42E-06
cancer	NUYTTEN_EZH2_TARGETS_UP	1.77E-05
leukemia	KRIGE_RESPONSE_TO_TOSEDOSTAT_24HR_UP	2.89E-05
cancer	CHARAFE_BREAST_CANCER_LUMINAL_VS_BASAL_DN	5.63E-05
leukemia	MULLIGHAN_MLL_SIGNATURE_2_UP	1.29E-04
cancer	GOZGIT_ESR1_TARGETS_DN	1.43E-04
leukemia	ALCALAY_AML_BY_NPM1_LOCALIZATION_UP	1.51E-04
cancer	KRIEG_HYPOXIA_NOT_VIA_KDM3A	2.33E-04
lymphocyte response to oncogene	DIRMEIER_LMP1_RESPONSE_LATE_UP	3.32E-04

Comparison of blood and non-blood samples show that the total set of distinguishing genes in cluster B are related to macrophage function and leukemia. (10 most strongly enriched gene sets shown.)

Supplementary Table 11: Enriched gene sets for cluster C from comparison of blood and non-blood samples

annotation	geneset	qval
cancer	BERTUCCI_MEDULLARY_VS_DUCTAL_BREAST_CANCER_DN	9.97E-04
cancer resistance	MASSARWEH_TAMOXIFEN_RESISTANCE_UP	9.97E-04
cancer	RODRIGUES_THYROID_CARCINOMA_ANAPLASTIC_DN	1.21E-02
cancer	KIM_WT1_TARGETS_12HR_UP	1.67E-02
T-cell lymphoma	PICCALUGA_ANGIOIMMUNOBLASTIC_LYMPHOMA_UP	1.67E-02
cancer	KRIEG_HYPOXIA_NOT_VIA_KDM3A	1.67E-02
mammary stem cells	LIM_MAMMARY_STEM_CELL_UP	2.70E-02
adipocyte differentiation	TSENG_ADIPOGENIC_POTENTIAL_DN	3.28E-02
cancer	GRAESSMANN_APOPTOSIS_BY_SERUM_DEPRIVATION_DN	3.28E-02
cancer	DODD_NASOPHARYNGEAL_CARCINOMA_UP	3.28E-02

Comparison of blood and non-blood samples show that the total set of distinguishing genes in cluster C are related to lymphoma and other cancers. (10 most strongly enriched gene sets shown.)

Supplementary Table 12: Enriched gene sets for cluster D from comparison of blood and non-blood comparison

annotation	geneset	qval
membrane	PLASMA_MEMBRANE	2.27E-14
membrane	INTEGRAL_TO_MEMBRANE	4.40E-14
membrane	INTRINSIC_TO_MEMBRANE	4.40E-14
membrane	PLASMA_MEMBRANE_PART	2.46E-13
membrane	MEMBRANE_PART	1.51E-12
membrane	INTEGRAL_TO_PLASMA_MEMBRANE	1.78E-12
membrane	INTRINSIC_TO_PLASMA_MEMBRANE	1.78E-12
membrane	MEMBRANE	2.11E-12
liver	HSIAO_LIVER_SPECIFIC_GENES	1.11E-09
MAP kinase	YOSHIMURA_MAPK8_TARGETS_UP	8.39E-09

Comparison of blood and non-blood samples reveal membrane function for gene cluster D. (10 most strongly enriched gene sets shown.)

Supplementary Table 13: Enriched gene sets for adult and fetal comparison

annotation	geneset	qval
liver	HSIAO_LIVER_SPECIFIC_GENES	1.47E-06
cytokine	KEGG_CYTOKINE_CYTOKINE_RECEPTOR_INTERACTION	1.47E-06
liver	CHIANG_LIVER_CANCER_SUBCLASS_PROLIFERATION_DN	6.07E-06
liver	YAMASHITA_LIVER_CANCER_STEM_CELL_DN	7.18E-05
MAPK8 (proliferation)	YOSHIMURA_MAPK8_TARGETS_UP	7.18E-05
H3K27me3	BENPORATH_ES_WITH_H3K27ME3	1.51E-04
liver	HOSHIDA_LIVER_CANCER_SUBCLASS_S3	2.21E-04
Polycomb targets	BENPORATH_EED_TARGETS	5.81E-04
Polycomb targets	BENPORATH_PRC2_TARGETS	2.83E-03
liver	SU_LIVER	4.88E-03

Comparison of adult and fetal samples show that distinguishing genes are related to liver and cytokine function. (10 most strongly enriched gene sets shown.)

Supplementary Table 14: Enriched gene sets for cluster A from comparison of adult and fetal samples

annotation	geneset	qval
apoptosis	GRAESSMANN_APOPTOSIS_BY_DOXORUBICIN_DN	2.12E-16
immune	MARSON_BOUND_BY_FOXP3_UNSTIMULATED	4.46E-13
erythroid differentiation from fetal liver	PILON_KLF1_TARGETS_DN	1.15E-09
apoptosis	GRAESSMANN_APOPTOSIS_BY_DOXORUBICIN_UP	2.29E-09
cancer (leukemia)	DIAZ_CHRONIC_MEYLOGENOUS_LEUKEMIA_UP	8.38E-09
anticancer treatment	BUYTAERT_PHOTODYNAMIC_THERAPY_STRESS_UP	1.07E-08
immune	MARSON_BOUND_BY_FOXP3_STIMULATED	4.54E-08
immune	GALINDO_IMMUNE_RESPONSE_TO_ENTEROTOXIN	1.86E-07
cancer (liver tumor)	ACEVEDO_LIVER_TUMOR_VS_NORMAL_ADJACENT_TISSUE_UP	1.36E-06
Alzheimer's	BLALOCK_ALZHEIMERS_DISEASE_UP	1.36E-06

Comparison of adult and fetal samples show that distinguishing genes in cluster A are related to fetal liver differentiation, immune response, and Alzheimer's disease. (10 most strongly enriched gene sets shown.)

Supplementary Table 15: Enriched gene sets for cluster B from comparison of adult and fetal samples

annotation	Geneset	qval
H3K27me3	BENPORATH_ES_WITH_H3K27ME3	8.94E-25
polycomb targets	BENPORATH_PRC2_TARGETS	2.30E-19
polycomb targets	BENPORATH_SUZ12_TARGETS	6.19E-18
polycomb targets	BENPORATH_EED_TARGETS	1.12E-17
MAP kinase	YOSHIMURA_MAPK8_TARGETS_UP	3.57E-17
H3K27me3, HCP	MEISSNER_BRAIN_HCP_WITH_H3K4ME3_AND_H3K27ME3	3.29E-16
liver	HSIAO_LIVER_SPECIFIC_GENES	3.67E-15
H3K27me3, HCP	MIKKELSEN_MEF_HCP_WITH_H3K27ME3	1.50E-14
membrane	INTRINSIC_TO_PLASMA_MEMBRANE	2.67E-14
membrane	PLASMA_MEMBRANE_PART	1.08E-13

Comparison of adult and fetal samples show that distinguishing genes in cluster B are related to polycomb targets and H3K27me3 modifications. (10 most strongly enriched gene sets shown.)

Supplementary Table 16: ChromDiff can capture epigenomic differences even when there are no differentially expressed genes

	PrimaryCulture vs PrimaryCell	PrimaryCell vs PrimaryTissue	Solid vs Liquid	PrimaryCulture vs PrimaryTissue
Distinguishing genes that are diff. expressed	0	0	0	129
Total distinguishing genes	198	939	3459	13727
Not distinguishing genes that are diff. expressed	0	0	0	178
Total not distinguishing genes	19546	18805	16285	6017

In three comparisons that yield no differentially expressed genes, ChromDiff still identifies genes showing epigenomic differences. These are three out of the four comparisons that still yield epigenomically distinguishing results when ChromDiff is limited to epigenomes with expression data. (This analysis used only epigenomes with expression data that was corrected for the same covariates as the chromatin state data. Any comparisons that yielded no significant ChromDiff differences are excluded.)

Supplementary Table 17: ChromDiff identifies chromatin state differences for Adult/Fetal, Female/Male, and CellLine/PrimaryCulture comparisons, while dPCA does not

Biological comparison	number of genes (dPCA)	number of genes (ChromDiff)
Adult_Fetal	0	5852
BRAIN_ESC	6488	7059
BRAIN_GI	4966	5533
BRAIN_MUSCLE	3750	0
BRAIN_SKIN	10080	2286
CellLine_PrimaryCulture	0	39
ESC_GI	7232	10457
ESC_SKIN	7847	0
Female_Male	0	369
MUSCLE_ESC	6450	0
MUSCLE_GI	3863	0
MUSCLE_SKIN	3256	0
PrimaryCell_PrimaryTissue	18231	17109
PrimaryCulture_PrimaryCell	18476	17827
PrimaryCulture_PrimaryTissue	16580	15481
SKIN_GI	12244	6830
SOLID_LIQUID	18423	17001

We compare ChromDiff to dPCA, the other existing method for group-wise epigenomic comparisons, by applying both methods to the same Epigenome Roadmap dataset using the same group comparisons. Although dPCA is unable to identify any epigenomic differences, ChromDiff identifies genes with chromatin state changes in the cases of a) Adult vs Fetal, b) Cell Lines vs Primary Cultures, and c) Female vs Male, which is especially relevant as they span different biological properties of age, sample heterogeneity, and sex.
